# Efficacy and safety of different noninvasive ventilation strategies for postextubation respiratory support in Neonatal Respiratory Distress Syndrome: a systematic review and network meta-analysis

**DOI:** 10.3389/fped.2024.1435518

**Published:** 2024-11-15

**Authors:** Jiayi Yang, Hua Mei, Xiaoli Wang, Jie Zhang, Mengyue Huo, Chun Xin

**Affiliations:** Department of Neonatology, Affiliated Hospital, Inner Mongolia Medical University, Hohhot, China

**Keywords:** noninvasive ventilation, NHFOV, NIPPV, NCPAP, N-BiPAP, NRDS

## Abstract

**Objective:**

The study aimed to compare the efficacy and safety of different noninvasive ventilation (NIV) modalities as primary respiratory support following extubation in Neonatal Respiratory Distress Syndrome (NRDS).

**Methods:**

A search was conducted in PubMed, Embase, Cochrane, Web of Science, China National Knowledge Network (CNKI), Wanfang database, VIP, and Chinese Biomedical Literature databases with a search time limit of April 2024 for the year of construction, and included randomized controlled clinical trials of different modes of noninvasive respiratory support after extubation in NRDS. The primary outcome indicators were the need for re-tracheal intubation within 72 h of extubation on noninvasive ventilatory support and carbon dioxide retention (PCO2) 24 h after extubation. Secondary outcome indicators included the incidence of bronchopulmonary dysplasia (BPD), nasal injury, pneumothorax, intraventricular hemorrhage (IVH) or periventricular white matter softening (PVL), retinopathy of prematurity (ROP), necrotizing enterocolitis (NEC), and mortality rate. A systematic review and network meta-analysis of the literature was performed by two investigators who screened, extracted, and evaluated the quality of the data. A systematic review and network meta-analysis were then performed using R software.

**Results:**

A total of 23 studies involving 2,331 neonates were analyzed. These studies examined four noninvasive respiratory modalities: continuous positive airway pressure ventilation (NCPAP), noninvasive intermittent positive pressure ventilation (NIPPV), bi-level positive airway pressure ventilation (N-BiPAP), and noninvasive high-frequency oscillatory ventilation (NHFOV). Results indicated that NHFOV, NIPPV, and N-BiPAP were significantly more effective than NCPAP in reducing the risk of reintubation (all *P* < 0.05), with NHFOV being the most effective. For carbon dioxide clearance, NHFOV outperformed both NIPPV and NCPAP (*P* < 0.05). Regarding the reduction of bronchopulmonary dysplasia (BPD) incidence, NHFOV and NIPPV showed a significant advantage over NCPAP.

**Conclusions:**

This network meta-analysis (NMA) suggested that NHFOV is the most effective mode of noninvasive respiratory support post-extubation, while NCPAP is the least effective. However, these findings should be interpreted with caution due to the limited number and quality of the studies included.

**Systematic Review Registration:**

https://www.crd.york.ac.uk/PROSPERO/#recordDetails, identifier (CRD42024544886).

## 1 Introduction

Neonatal Respiratory Distress Syndrome (NRDS) is a prevalent respiratory crisis in neonatology, which is defined as acute respiratory failure with extensive alveolar atrophy damage exudation in both lungs due to pulmonary surfactant (PS) deficiency (1, 2), manifesting as progressive dyspnoea, cyanosis, and respiratory failure within the first few hours after birth, predominantly in preterm infants. The incidence of NRDS increases with decreasing gestational age. Treatment primarily involved postnatal mechanical ventilation using an autonomous ventilator to enhance the respiratory status of the neonate. In recent years, the use of invasive mechanical ventilation (IMV) has significantly enhanced survival rates in NRDS, however, it also heightens the risk of ventilator-associated complications, particularly among infants requiring repeated intubations or prolonged periods of IMV. Consequently, to enhance survival quality, early extubation to noninvasive respiratory support (NRS) has become a critical focus (3). NRS modalities commonly utilized in neonatal care include continuous positive airway pressure ventilation (NCPAP), noninvasive intermittent positive pressure ventilation (NIPPV), bi-level positive airway pressure ventilation (N-BiPAP), and noninvasive high-frequency oscillatory ventilation (NHFOV). NCPAP delivers a constant positive pressure to the airways, primarily through a nasal cannula or mask, to maintain alveolar expansion and prevent collapse. In contrast, NIPPV adds periodic bursts of higher pressure on top of the baseline CPAP, aiding the infant's spontaneous breathing, increasing tidal volume, and enhancing overall ventilation. N-BiPAP (Nasal Bi-level Positive Airway Pressure) offers two distinct pressure levels—a higher pressure during inspiration (IPAP) and a lower pressure during expiration (EPAP). This approach improves ventilation and oxygenation, making it particularly effective for infants who need extra inspiratory support. NHFOV is a newer noninvasive support method that delivers rapid, small breaths through high-frequency oscillations, helping to keep the airways open and improve carbon dioxide clearance (4–6). The physiological mechanisms underlying these NRS modalities vary significantly, and their relative merits remain under debate (7). Although numerous systematic reviews have compared the effects of these modalities on post-extubation ventilatory support, a comprehensive evaluation through a network meta-analysis (NMA) has not yet been conducted. Therefore, this systematic review and NMA aimed to assess the effectiveness and safety of various NRS modalities—NCPAP, NIPPV, N-BiPAP, and NHFOV-through a network meta-analysis. Effectiveness was assessed primarily by successful extubation rates and reduced reintubation rates, while safety focused on the incidence of ventilator-related complications such as bronchopulmonary dysplasia, nasal injury, air leaks, and cerebral hemorrhage. This study aimed to provide an evidence-based basis for selecting the optimal mode of noninvasive ventilation after extubation in preterm infants.

## 2 Methods

### 2.1 Register

In this study, we compared the efficacy and safety of various noninvasive ventilation strategies for respiratory support post-extubation in NRDS, utilizing a systematic evaluation protocol registered in the PROSPERO registry (CRD42024544886). This review adhered to the PRISMA guidelines for network meta-analyses (8).

### 2.2 Search strategy

A comprehensive search was conducted for clinical randomized controlled trials on NRDS in databases including PubMed, Embase, Cochrane, Web of Science, CNKI, Wanfang Database, VIP, and China Biomedical Literature Database from their inception until April 2024. This search focused on trials that adhered to the established diagnostic criteria for NRDS and explored noninvasive ventilation strategies. The search was guided by the PICOS framework: (P) Population: neonates diagnosed with NRDS; (I) Intervention: noninvasive ventilation strategies; (C) Comparison: the effectiveness and safety of NHFOV, NIPPV, NCPAP, and N-BiPAP in respiratory support for neonates post-extubation; (O) Outcome: 1. The primary outcome: re-tracheal intubation within 72 h of extubation on noninvasive ventilatory support; 2. carbon dioxide retention (PCO2) 24 h after extubation. Secondary outcome: the incidence of bronchopulmonary dysplasia (BPD), nasal injury, pneumothorax, intraventricular hemorrhage (IVH) or periventricular white matter softening (PVL), retinopathy of prematurity (ROP), necrotizing enterocolitis (NEC) and mortality rate; (S) Study Type: Randomized controlled trials. Details of the search terms are available in the Additional Materials section (Supplementary Table S1).

### 2.3 Inclusion criteria and exclusion criteria

Inclusion criteria: (1) Study subjects: neonates aged 0–28 days postnatally; (2) diagnosed with NRDS (9): 1. Clinical manifestations: These include shortness of breath (>60 breaths/min), nasal flaring, expiratory groaning, subpectoral or intercostal depression, and bruising. 2. Chest imaging: chest radiographs show typical “hairy glass-like” changes, accompanied by air bronchial signs. 3. Blood gas analysis: hypoxaemia (PaO2 < 50 mmHg) with or without metabolic or respiratory acidosis. 4. Preterm labour: gestational age < 37 weeks, and the risk of RDS is higher the earlier the birth; (3) initially intubated and provided invasive ventilatory support for NRDS, followed by noninvasive support upon meeting extubation standards. Exclusion criteria: (1) studies lacking clear diagnostic criteria; (2) studies involving congenital anomalies (e.g., congenital diaphragmatic hernia, congenital lung anomalies, congenital heart disease, except for patent foramen ovale and arterial ductus arteriosus) or infectious shock; (3) prophylactic, retrospective studies, and case reports; (4) studies with unclear endpoints or those that failed to provide valid data for analysis; (5) studies published multiple times.

### 2.4 Noninvasive ventilation modes categories

Ventilation strategies in the included randomized controlled trials were classified into four models:

1. NHFOV; 2. NIPPV; 3. NCPAP; 4. N-BiPAP

The parameter settings for each mode, including settings used during ventilation and those applied after meeting extubation criteria (Table 1).

**Table 1:** Characteristics of the included studies.

	Author	Gestational age (wk)	Birth weight	IMV	Surfactants	Initial parameter settings	Samplesize	Interventions	Primary outcome	Secondary outcome
1	Ahmed 2023 (10) (Egypt)	NIPPV (33.40 ± 2.06) NHFOV (33.97 ± 1.65)	NIPPV (2,050 ± 590) NHFOV (2,280 ± 520)	No mention	Curosurf, 200 mg/kg	NHFOV (Frequency: 8–12 Hz, Amplitude: 25–40; FiO2:0.21–0.40) NIPPV (PIP: 15–25 cmH2O, PEEP: 5–10 cmH2O; FiO2 0.21–0.40)	60 (30,30)	NHFOV vs. NIPPV	1.72 h re-intubation 2. After extubation PCO2	①②④⑥⑦
2	Yuan 2022 (11) (China)	NIPPV (30.38 ± 1.61) NHFOV (30.60 ± 1.71) NCPAP (30.12 ± 1.74)	NIPPV (1,420 ± 300) NHFOV (1,037 ± 330) NCPAP (1,380 ± 280)	ASSC	Curosurf, dose unknown	NHFOV (MAP: 6–12 cmH2O RR 6–12 Hz FiO2: 0.3–0.5) NIPPV (PIP: 20–25 cmH2O, PEEP: 5–6 cmH2O; FiO2 0.3–0.5 RR 25–30 times/min) NCPAP (FiO2: 0.3–0.5; PEEP:4–6 cmH2O)	120 (40,40,40)	NHFOV vs. NIPPV vs. NCPAP	1.72 h re-intubation	①②③④⑥
3	El-Farrash 2022 (12) (USA)	NIPPV (32.70 ± 1.60) N-BiPAP (32.07 ± 1.86) NCPAP (32.85 ± 1.37)	NIPPV (1,940 ± 450) N-BiPAP (1,700 ± 440) NCPAP (1,810 ± 420)	SIMV	Survanta, 200 mg/kg	NIPPV (PEEP: 4–6 cmH2O; PIP:13–17 cmH2O) NCPAP (PEEP: 4–8 cmH2O) N-BiPAP (IPAP: 5 cmH2O, EPAP:9 cmH2O)	120 (40,40,40)	NIPPV vs. NCPAP vs. N-BiPAP	1.48 h re-intubation	③④
4	Liu 2021 (13) (China)	NHFOV (29.24 ± 1.60) NCPAP (29.83 ± 1.71)	NHFOV (1,220 ± 300) NCPAP (1,330 ± 330)	SIMV	No mention	NHFOV (MAP: 12∼15 cmH2O, FiO2:30%–40%, Frequency:12∼15hz, Amplitude: 25∼30 cmH20) NCPAP (FiO2: 30%–40%, PEEP: 6 cmH2O, FLOW: 6–8 L/min)	68 (34,34)	NHFOV vs NCPAP	1.72 h re-intubation 2.24 h. After extubation PCO2	①④⑤
5	Seth 2021 (14) (India)	NIPPV 31 (29–35) NHFOV 32 (28–35)	NIPPV 1,495 (980–2,214) NHFOV 1,500 (1,120–2,140)	SIMV	No mention	NHFOV (MAP: 8–10 cmH2O, Frequency: 10–12 hz, Amplitude: 25–35 cmH2O) NIPPV (PIP: PIP: 2 cm H2O higher than pre-intubation, PEEP: 4–6 cmH2O RR 40 times/min)	86 (43,43)	NHFOV vs NIPPV	1.72 h re-intubation 2.12 h. After extubation PCO2	①③④
6	Pan 2021 (15) (China)	N-BiPAP (30.1 ± 1.8) NCPAP (29.6 ± 2.0)	N-BiPAP (1,251 ± 158) NCPAP (1,264 ± 152)	No mention	No mention	NCPAP (6–cmH2O) N-BiPAP (9/5–cmH2O)	284 (N-BiPAP144. NCPAP140)	NCPAP vs. N-BiPAP	1.72 h re-intubation	①④⑤⑥
7	Jia 2021 (16) (China)	NIPPV (31.77 ± 1.50) NHFOV (31.89 ± 1.42)	NIPPV (1,650 ± 400) NHFOV (1,680 ± 350)	No mention	No mention	NHFOV (Frequency: 6–12 Hz; Amplitude: 12–16 cmH2O; MAP: 6–8 cmH2O; FiO2: 0.25–0.40) NIPPV (PIP: 17–27 cmH2O, PEEP: 4–6 cmH2O; RR:15–40 times/min)	100 (50,50)	NHFOV vs. NIPPV	1.72 h re-intubation 2.24 h After extubation PCO2	①②③④
8	Malakian 2021 (17) (Iran)	NCPAP (31.13 ± 1.77) N-BiPAP (31.32 ± 1.53)	NCPAP (1,415 ± 233.15) N-BiPAP (1,377.91 ± 260.24)	INSURE	Survanta, 100 mg/kg	NCPAP (FiO2: 40%, PEEP: 5 cmH2O) N-BiPAP (FiO2: 40%, *P* peak voltage high: 8 cmH20, *P*-peak depression value:5 cmH2O)	148 (74, 74)	NCPAP vs. N-BiPAP	1.72 h re-intubation	①④⑦
9	Ding 2020 (18) (China)	NIPPV (29.7 ± 2.3) NCPAP (29.9 ± 1.4)	NIPPV (1,300 ± 200) NCPAP (1,100 ± 200)	No mention	No mention	NIPPV (PIP: 15–25 cmH2O, PEEP: 4–6 cmH20, RR: 15–50 times/min) NCPAP (PEEP: 6 cmH2O)	80 (40,40)	NIPPV vs. NCPAP	1.72 h re-intubation	①⑤⑦
10	Feng 2020 (19) (China)	NHFOV (32.23 ± 1.42) NCPAP (32.43 ± 1.34)	NHFOV (1,957.63 ± 76.37) NCPAP (1,987.43 ± 96.21)	SIMV	Calsurf, 70 mg/kg	NHFOV (MAP: 6–10 cmH2O, Frequency: 5–10 Hz, FiO2: 0.3–0.4) NCPAP (FiO2: 30%–40%, PEEP: 4–6 cmH2O, FLOW: 6–10 L/min)	48 (24,24)	NHFOV vs. NCPAP	1.72 h re-intubation 2.24 h After extubation PCO2	③④
11	Li 2019 (20) (China)	NCPAP (28.77 ± 3.01) N-BiPAP (28.39 ± 2.98)	NCPAP (1,320.91 ± 179.55) N-BiPAP (1,323.29 ± 173.02)	SIMV	No mention	NCPAP (FiO2: 0.3%–0.4%, PEEP: 4–6 cmH2O, FLOW: 6–8 L/min) N-BiPAP (FiO2: 0.3%–0.4%, PEEP: 4–6 cmH2O, PIP: 10–15 cmH2O)	52 (26,26)	NCPAP vs. N-BiPAP	1.72 h re-intubation 2.24 h. After extubation PCO2	①④⑥
12	Wang 2019 (21) (China)	NHFOV (29.7 ± 1.2) NIPPV (29.6 ± 1.4)	NHFOV (1,270 ± 115) NIPPV (1,265 ± 120)	SIMV	No mention	NHFOV (FiO2 25%–40%, MAP: 6–8 cmH2O, Amplitude: 12–16 cmH2O, Frequency: 6–10 Hz) NIPPV (PIP: 15–20 cmH2O, PEEP: 4–6 cmH2O, FiO2 25%∼40%, RR: 35–50 times/min)	103 (NIPPV53. NHFOV50)	NHFOV vs. NIPPV	1.72 h re-intubation	①②③④⑤
13	Chen 2019 (22) (China)	NCPAP (32.09 ± 1.53) NIPPV (32.09 ± 1.53)	NCPAP (1,842.8 ± 292.3) NIPPV (1,831.3 ± 2,588.6)	INSURE	Curosurf, 100 mg/kg	NIPPV (PIP: 15–25 cmH2O, PEEP: 4–6 cmH2O, FiO2: 0.21–0.40) NCPAP (4–6 cmH2O, FiO2: 0.21–0.40)	286 (143, 143)	NIPPV vs. NCPAP	1.72 h re-intubation	①③④⑤⑥⑦
14	Najafian 2019 (23) (Iran)	NCPAP (31.73 ± 1.72) NIPPV (32.6 ± 1.92)	NCPAP (1,490 ± 265.09) NIPPV (1,529.3 ± 225.7)	No mention	No mention	No mention	60 (30,30)	NIPPV vs. NCPAP	1.72 h re-intubation 2.24 h. After extubation PCO2	①④⑥
15	Lou 2018 (24) (China)	NHFOV (33.5 ± 1.5) BiPAP (34.2 ± 1.6)	NHFOV (1.79 ± 0.33) BiPAP (1.84 ± 0.42)	INSURE	Curosurf, 200 mg/kg	NHFOV (FiO2: 0.30–0.40, Frequency: 6–12 Hz, MAP: 6–12 cmH2O) BiPAP (FiO2: 0.3–0.4, PEEP: 5 cmH2O, PIP:12–15 cmH2O)	55 (NHFOV33. BiPAP 32)	NHFOV vs. N-BiPAP	1.72 h re-intubation 2.24 h. After extubation PCO2	①③④⑥⑦
16	Santos 2017 (25) (Brazil)	NCPAP (29.58 ± 1.96) NIPPV (29.28 ± 1.7)	NCPAP (1,161.64 ± 225.07) NIPPV (1,121.53 ± 232.81)	No mention	Curosurf, 200 mg/kg	NIPPV (PIP: 14–16 cm H2O, PEEP 4–6 cm H2O, RR: 12–18 times/min) NCPAP (PEEP 4–5 cmH2O, FLOW: 6–7 L/min)	69 (NCPAP33. NIPPV36)	NIPPV vs. NCPAP	1.72 h re-intubation 2.24 h. After extubation PCO2	①④⑤⑥⑦
17	Esmaeilnia2016 (26) (Iran)	NCPAP (32.15 ± 2.03) NIPPV (32.04 ± 2.91)	NCPAP (1,627 ± 539) NIPPV (1,637 ± 631)	INSURE	Survanta, 100 mg/kg	No mention	150 (NCPAP73. NIPPV77)	NIPPV vs. NCPAP	1.72 h re-intubation	①④⑥
18	Jasani 2016 (27) (Iran)	NCPAP (30.6 ± 2.6) NIPPV (30.8 ± 2.7)	NCPAP (1,153 ± 283) NIPPV (1,187 ± 310)	PSV	Survanta/Neosurf, dose unknown	NIPPV (4 cm H2O higher than pre-intubation) CPAP (5–6 cm H2O)	63 (NCPAP32. NIPPV31)	NIPPV vs. NCPAP	1.72 h re-intubation	①④⑤⑦
19	Farhat 2015 (28) (Iran)	NCPAP (31.1 ± 2) NIPPV (31.8 ± 1.7)	NCPAP (1,650 ± 486) NIPPV (1,622 ± 437)	INSURE	Currosurf/Survanta, 100 mg/kg	NIPPV (PIP: 18–20 cmH2O, PEEP: 4–5 cmH2O, FiO2: 0.21–0.5) NCPAP (PEEP:6cmH2O)	106 (53,53)	NIPPV vs. NCPAP	1.72 h re-intubation	①②③⑤
20	Kong 2014 (29) (China)	NCPAP (32.4 ± 1.4) N-BiPAP (32.5 ± 1.4)	NCPAP (1,975 ± 553) N-BiPAP (1,954 ± 560)	SIMV/A/C	Calsurf, 70 mg/kg	N-BiPAP (PIP: 12–15 cmH2O, PEEP: 4–6 cmH2O, FiO2: 0.2–0.3, Frequency: 15–30 times/min) NCPAP (PEEP: 4–6 cmH2O, FiO2: 0.30–0.45, Flow 6–8 L/min)	69 (BiPAP35. CPAP34)	NCPAP vs. N-BiPAP	1.72 h re-intubation 2.24 h. After extubation PCO2	①④⑤⑥⑦
21	Kahramaner 2014 (30) (Turkey)	NCPAP (28.3 ± 2.1) NIPPV (29.3 ± 3.0)	NCPAP (1,091 ± 298) NIPPV (1,228 ± 429)	SIPPV/SIMV	Survanta, 100 mg/kg	NIPPV (PIP:2 cm H2O higher than pre-intubation, PEEP: 6 cm H2O, FiO2: 0.4) PEEP (6 cmH2O, FiO2: 0.4)	67 (NCPAP28. NIPPV39)	NIPPV vs. NCPAP	1.48 h re-intubation	①④⑤⑥⑦
22	Moretti 2008 (31) (Rome)	NCPAP (27.1 ± 2.6) NIPPV (26.9 ± 1.7)	NCPAP (957 ± 213) NIPPV (908 ± 192)	A/C/PAV	No mention	NIPPV (PIP:10–20 cmH2O, FLOW:6–8 L/min) NCPAP (PEEP:3–5 cmH2O, FLOW: 6–10 L/min)	63 (NCPAP31. NIPPV32)	NIPPV vs. NCPAP	1.72 h re-intubation	②③④⑤⑥⑦
23	Khalaf 2001 (32) (USA)	NCPAP (27.6 ± 0.6) NIPPV (27.7 ± 0.6)	NCPAP (1,032 ± 76) NIPPV (1,088 ± 64)	No mention	Survanta, dose unknown	NIPPV (PIP: 16–20 cmH2O, PEEP: 4–6 cmH2O, FiO2: 0.35–0.45) NCPAP (PEEP: 4–6 cmH2O, FiO2: 0.35–0.45)	64 (NCPAP30. NIPPV34)	NIPPV vs. NCPAP	1.72 h re-intubation	②③④⑤⑥⑦

① BPD; ② Nasal injuries; ③ Air leak; ④ IVH or PVL; ⑤ ROP; ⑥ NEC; ⑦ Mortality rate.

### 2.5 Study selection

In this study, two uniformly trained researchers independently performed the initial screening and selected studies based on the inclusion and exclusion criteria. In the event of a disagreement during the screening process, a third researcher resolved whether to include or exclude the studies.

### 2.6 Risk of bias of individual studies

To evaluate the quality of the included studies, this study employed the Cochrane Risk of Bias Tool for bias assessment (33), which assesses several criteria including randomized sequence generation, allocation concealment, blinding of participants and personnel, blinding of outcome assessment, incomplete outcome data, selective reporting, and other potential sources of bias. Trials were categorized into three levels based on the number of bias-prone components identified: high risk (five or more), some concerns (three or four), and low risk (two or fewer). Due to the impracticality of blinding participants in the ventilation strategy intervention protocol, all studies were deemed at high risk for “subject blinding,” this component was therefore excluded from the overall risk of bias evaluation.

### 2.7 Data extraction

Baseline data extracted for this study included: the first author, publication date, ventilation strategy, number of cases, gender, birth weight, gestational age, initial mode parameter settings, and data related to outcome indicators. The study incorporated four mechanical ventilation strategies: NCPAP, NIPPV, N-BiPAP, and NHFOV. Primary outcome indicators were the need for re-tracheal intubation within 72 h post-noninvasive respiratory support and PCO2 retention 24 h post-extubation. Secondary outcome indicators included the incidence of bronchopulmonary dysplasia (BPD), nasal injury, air leaks, intraventricular hemorrhage (IVH) or periventricular leukomalacia (PVL), retinopathy of prematurity (ROP), necrotizing enterocolitis (NEC) and mortality rate.

### 2.8 Statistical analysis

This study assessed the safety of each ventilation treatment strategy using risk ratios (RR) with 95% credible intervals (CrIs) addressing both dichotomous and continuous data to maximize accuracy. Literature that met the inclusion criteria was subjected to Cochrane bias assessment using Revman 5.3 software. Network geometry was evaluated using a network diagram in R software (Version-R 4.3.3) (34), where the size of the nodes reflects the number of subjects in each intervention, and the thickness of the lines between nodes indicates the number of studies in each comparison. Model convergence was assessed with Gelman-Rubin plots, trace, and density plots. Inconsistencies were examined through node splitting. Additionally, a paired meta-analysis of direct evidence across different modes of noninvasive respiratory support was conducted, and heterogeneity was assessed using the I^2^ statistic and the Cochran Q-test. The results of the NMA were presented as 95% CIs in league matrix tables and forest plots. The league matrix illustrates the RR of the intervention outcomes with rows vs. columns in the lower triangles and vice versa in the upper triangles. All interventions were ranked for outcomes using the Surface Under the Cumulative Ranking curve (SUCRA), an index ranging from 0 (least effective intervention) to 1 (most effective intervention). Interpretation of SUCRA should consider the 95% CrIs and the quality of evidence. The confidence in the final estimates for all the outcomes was assessed using the GRADE approach as recommended by the GRADE working group (8).

## 3 Results

### 3.1 Screening process for inclusion of studies

**Figure 1:**
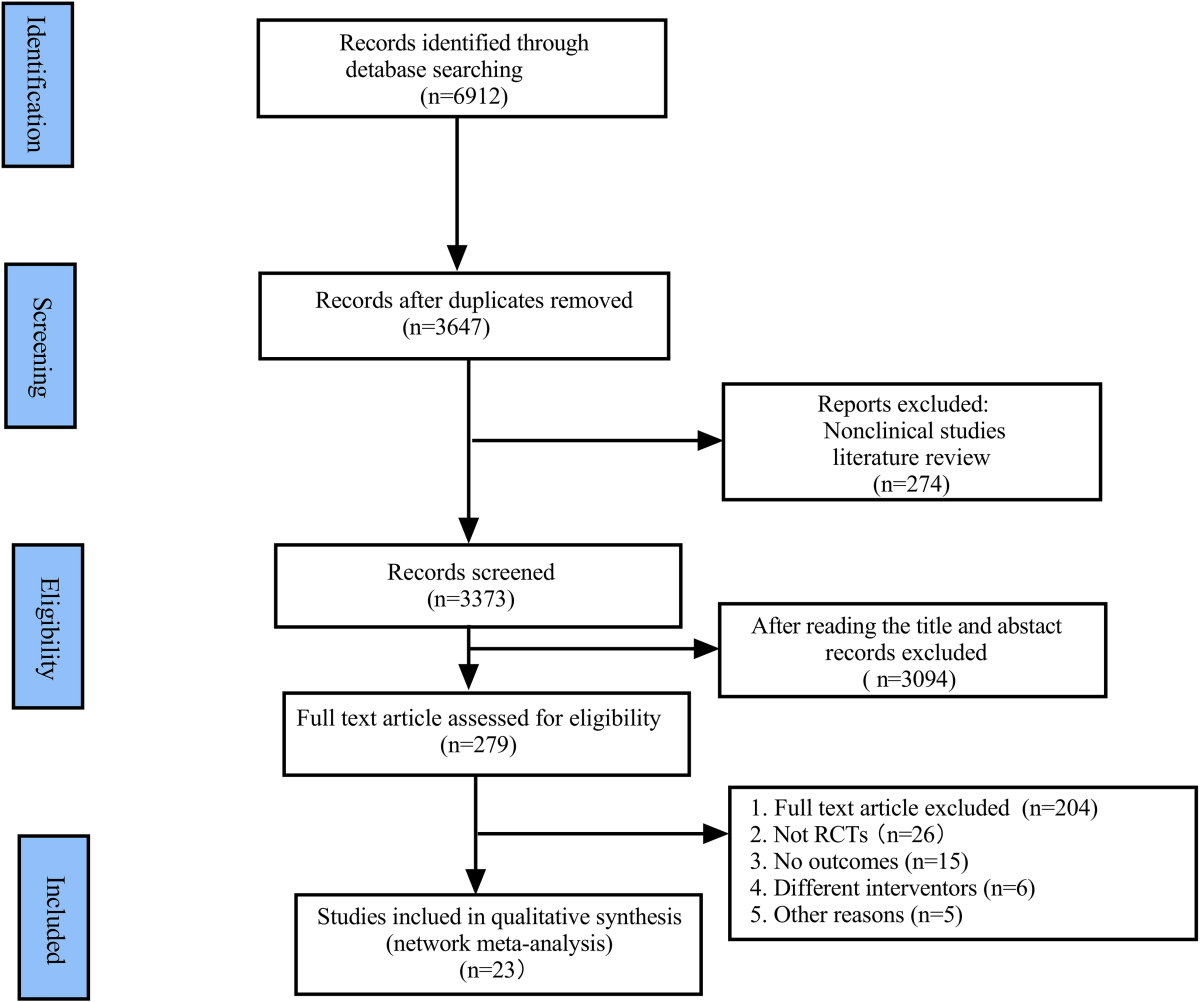
PRISMA flow chart.

A total of 6,912 studies were initially identified from various databases, with 23 studies ultimately included after excluding those for relevant reasons. The PRISMA flow chart is depicted in Figure 1.

### 3.2 Characteristics of the included studies

Table 1 outlines the general characteristics of the included studies, comprising 2,331 newborns across 23 studies, which include 21 two-armed trials and 2 three-armed trials. The geographic distribution of these trials included 17 in Asia, 2 in North America, 2 in Europe, 1 in South America, and 1 in Africa. Study durations varied from 1 year to over 3 years. The subjects were predominantly preterm infants diagnosed with NRDS at birth, with gestational ages ranging from 28 to 35 weeks. 14 studies involved neonates who had received surfactant treatment prior to randomization into subgroups. In the majority of these studies, treatment failures and reintubations occurred within 72 h of randomization. The specific NRS setup parameters employed are detailed in Table 1.

### 3.3 Risk of bias assessment of included studies

**Figure 2:**
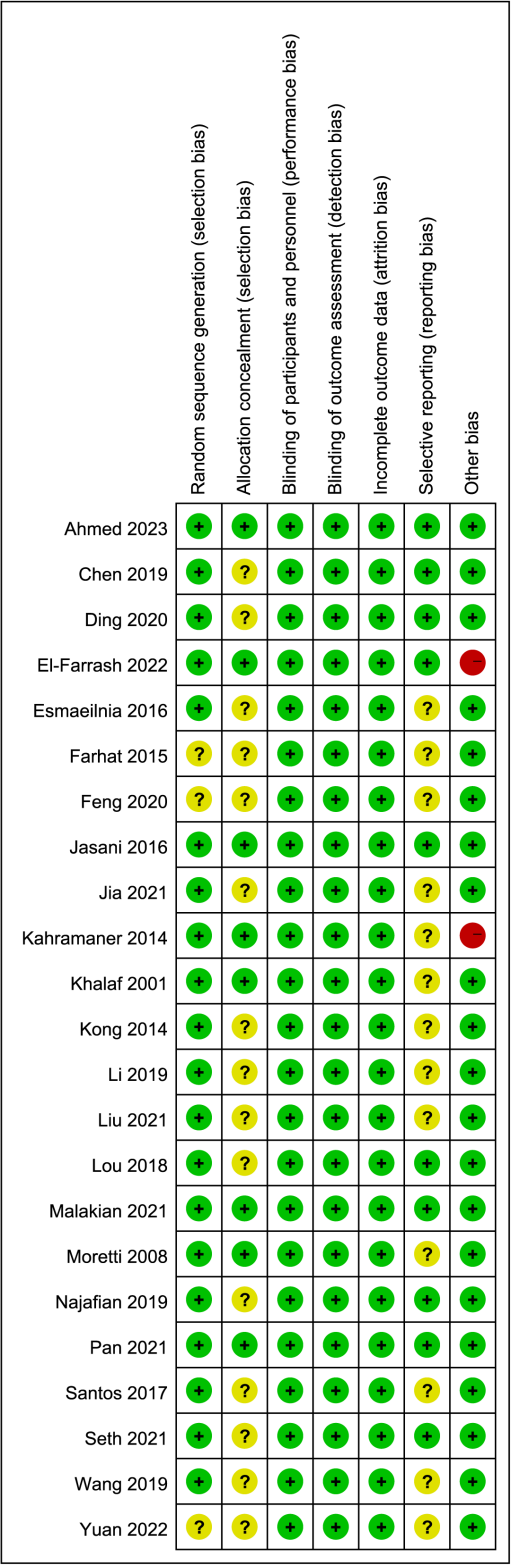
Risk of bias summary.

Three studies were assessed as having a moderate risk of bias, and 15 were considered to have a low risk of bias, primarily due to issues with randomized sequence generation and allocation concealment. Due to the nature of the interventions, it was impossible to blind personnel and outcome assessors in all trials. The overall risk of bias is illustrated in Figure 2.

### 3.4 Primary outcomes

#### 3.4.1 Rate of reintubation within 72 h after extubation

**Figure 3:**
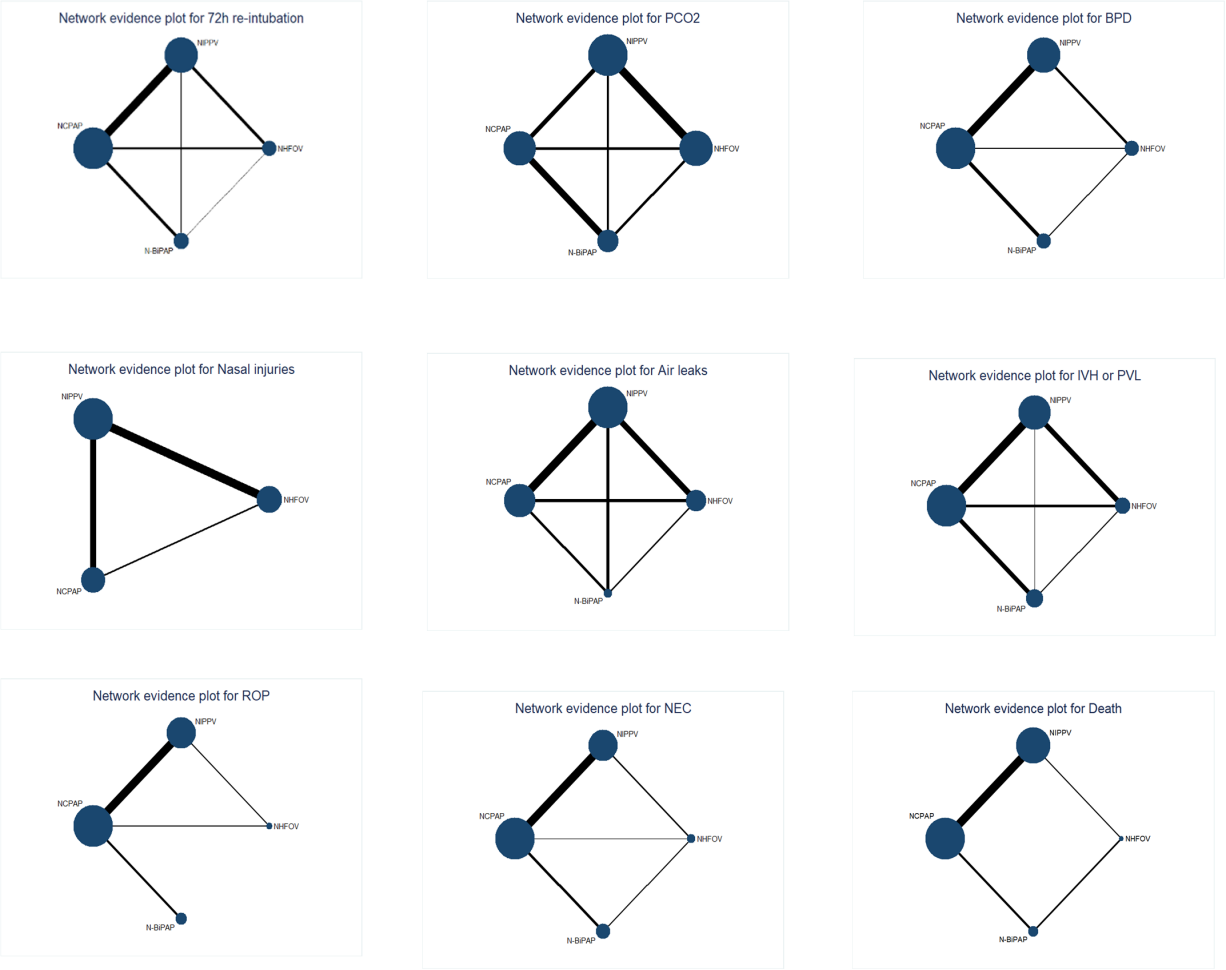
Geometry of the network for the outcomes. The size of the nodes reflects the number of studies and total number of patients involved in each intervention. The thickness of the edges indicates the number of trials directly compared.

**Figure 4:**
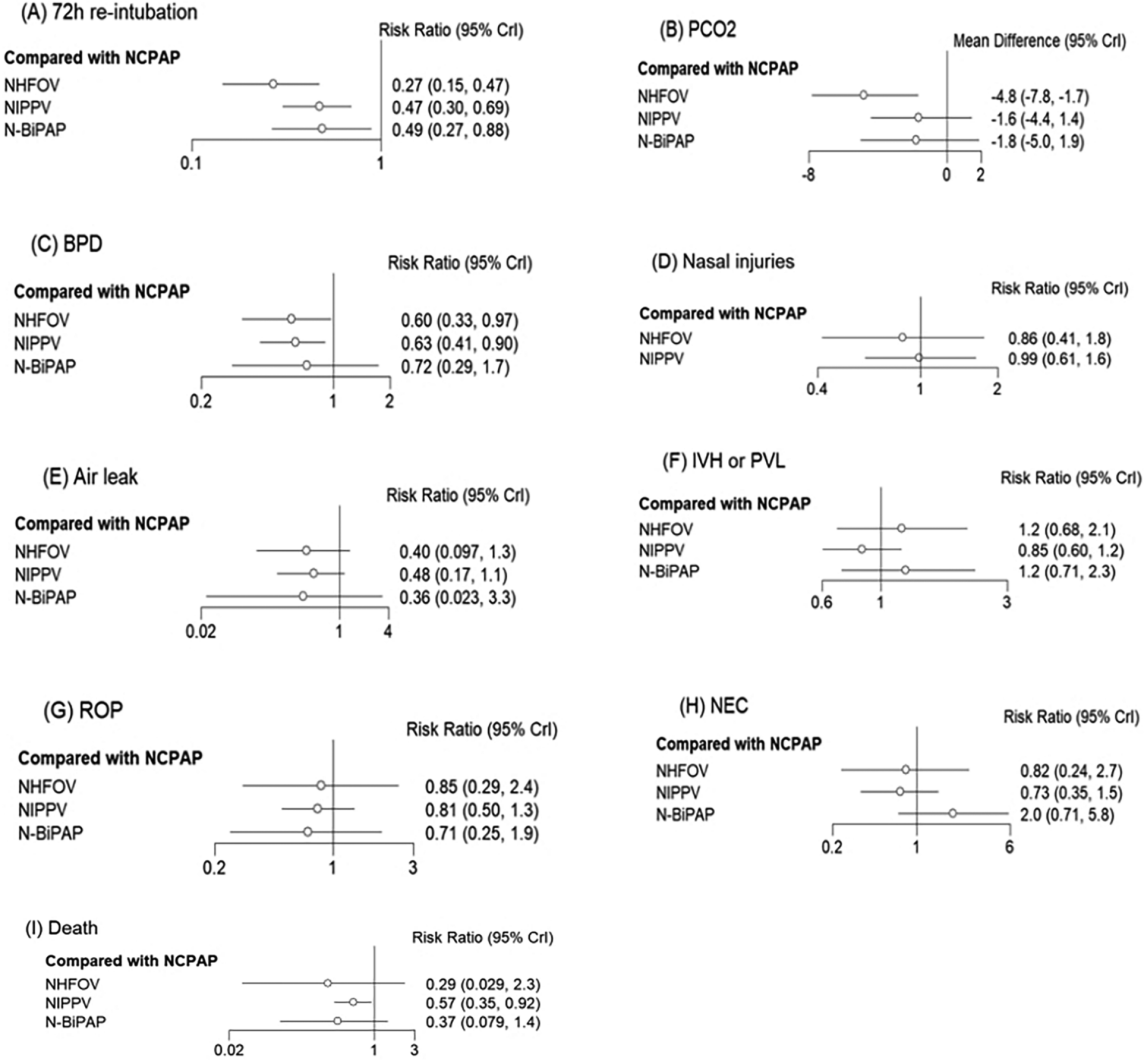
Forest plots depicting the RR (95% CrI) for different NRS modalities: **(A)** 72 h re-intubation, **(B)** PCO2, **(C)** BPD, **(D)** Nasal injuries, **(E)** Air leak, **(F)** IVH or PVL, **(G)** ROP, **(H)** NEC, **(I)** Death.

**Figure 5:**
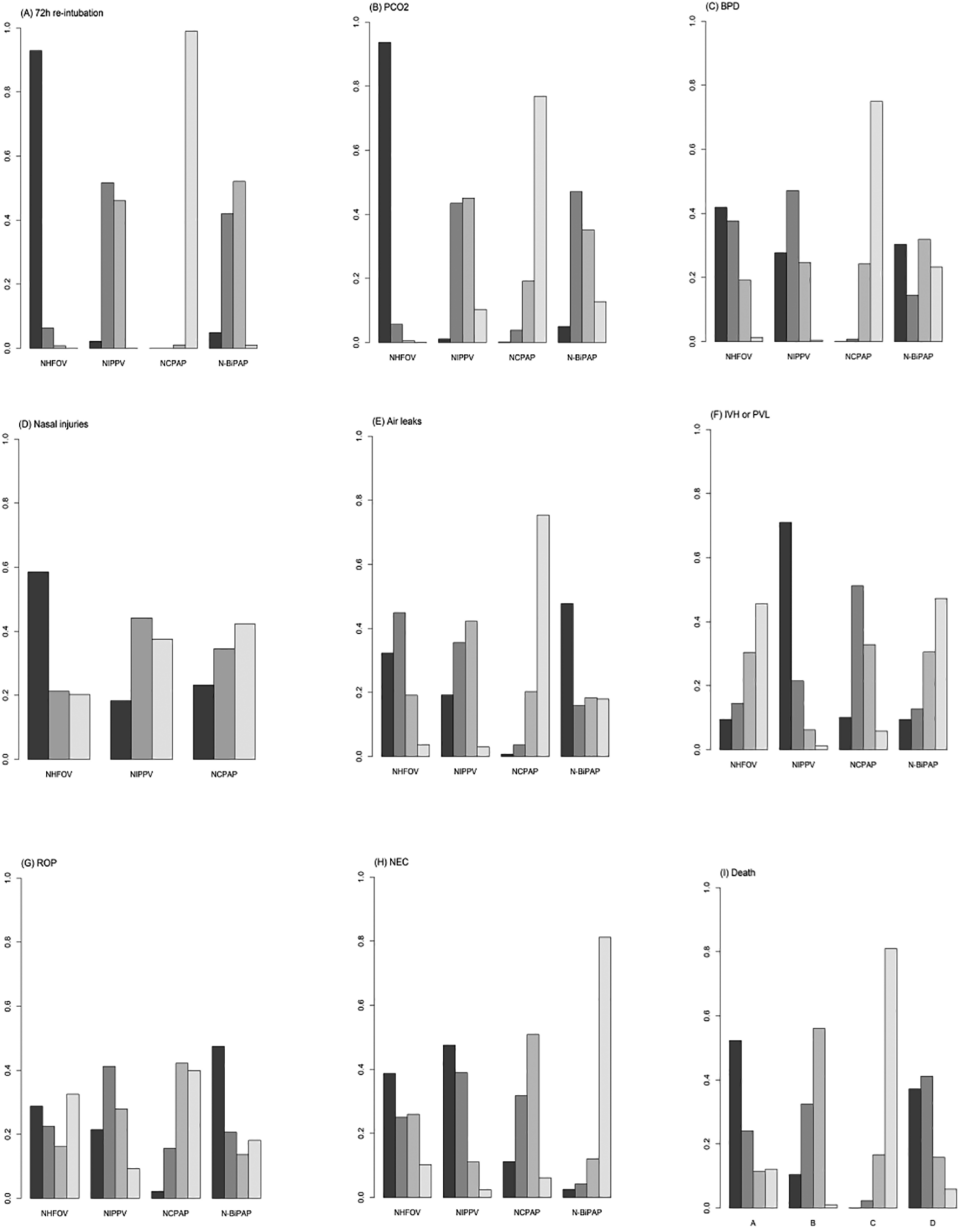
SUCRAs plots for the outcomes: **(A)** 72 h re-intubation, **(B)** PCO2, **(C)** BPD, **(D)** Nasal injuries, **(E)** Air leaks, **(F)** IVH or PVL, **(G)** ROP, **(H)** NEC, and **(I)** Death.

A total of 23 studies (10–32) involving 2,331 newborns were analyzed, documenting 417 events within the network (Table 2). Closed loops among all interventions were depicted in the network relationship diagram (Figure 3), with the majority of data, contributing 26.2%, coming from comparisons between NIPPV and NCPAP (Table 2; Supplementary Material). A local inconsistency test using node splitting indicated *P* > 0.05, and data were analyzed using a consistency model. Potential Scale Reduction Factor (PSRF) is a metric in Bayesian statistics used to assess the convergence of the Markov Chain Monte Carlo (MCMC) algorithm. The PSRF for all three outcome indicators converged to 1, suggesting robust convergence (Supplementary Material). Separate heterogeneity tests on the original studies with two or more contributions showed significant heterogeneity between NCPAP and N-BiPAP (*I*^2^ = 54.8), though still within acceptable limits; no significant heterogeneity was noted among the remaining studies. Network meta-analysis revealed that NHFOV [0.27 (0.15,0.47)], NIPPV [0.47 (0.30,0.69)], and N-BiPAP [0.49 (0.27,0.88)] significantly reduced the risk of reintubation compared to NCPAP (Figure 4; Table 3). The SUCRA values for the four ventilation modes were 0.97, 0.52, 0.00, and 0.50, respectively, ranking the effectiveness in reducing reintubation post-extubation from highest to lowest as NHFOV > NIPPV > N-BiPA*P* > NCPAP. This suggests that NHFOV was the most effective mode for post-extubation respiratory support, offering the greatest likelihood of reducing reintubation (Figure 5).

**Table 2:** Network characteristics.

Comparison	Number of study	Number of subjects	Number of outcomes	Event rate (%)
Primary outcome–rate of reintubation within 72 h after extubation				
NHFOV vs. NIPPV	5	429	74	24.5
NHFOV vs. NCPAP	3	196	36	10.9
NHFOV vs. N-BiPAP	1	65	19	14.2
NIPPV vs. NCPAP	12	1,168	195	26.2
NIPPV vs. N-BiPAP	1	80	8	5.6
NCPAP vs. N-BiPAP	5	633	85	18.6
Primary outcome - PCO2 level within 24 h after changing to noninvasive assisted ventilation				
NHFOV vs. NIPPV	4	349	42.23 ± 7.48	25.8
NHFOV vs. NCPAP	2	116	46.50 ± 4.58	16.8
NHFOV vs. N-BiPAP	1	65	40.85 ± 8.60	13.1
NIPPV vs. NCPAP	3	207	41.95 ± 5.55	16.7
NIPPV vs. N-BiPAP	1	80	45.55 ± 8.06	17.0
NCPAP vs. N-BiPAP	3	201	42.40 ± 7.64	10.6
Secondary outcomes - BPD				
NHFOV vs. NIPPV	5	429	80	28.4
NHFOV vs. NCPAP	2	148	46	3.5
NHFOV vs. N-BiPAP	1	65	2	3.8
NIPPV vs. NCPAP	11	1,168	138	36.0
NCPAP vs. N-BiPAP	4	553	26	28.3
Secondary outcomes - Nasal injuries				
NHFOV vs. NIPPV	4	240	29	36.8
NHFOV vs. NCPAP	1	80	19	21.4
NIPPV vs. NCPAP	4	371	66	41.8
Secondary outcomes - Air leak				
NHFOV vs. NIPPV	4	369	19	24.0
NHFOV vs. NCPAP	2	128	15	16.1
NHFOV vs. N-BiPAP	1	65	5	20.9
NIPPV vs. NCPAP	6	679	54	24.7
NIPPV vs. N-BiPAP	1	80	0	5.3
NCPAP vs. N-BiPAP	1	80	2	9.0
Secondary outcomes - IVH or PVL				
NHFOV vs. NIPPV	5	429	33	18.7
NHFOV vs. NCPAP	3	196	30	16.6
NHFOV vs. N-BiPAP	1	65	3	3.0
NIPPV vs. NCPAP	10	942	174	29.2
NIPPV vs. N-BiPAP	1	80	2	5.1
NCPAP vs. N-BiPAP	5	593	59	27.5
Secondary outcomes - ROP				
NHFOV vs. NIPPV	1	103	2	12.4
NHFOV vs. NCPAP	1	68	13	22.0
NIPPV vs. NCPAP	8	798	100	33.7
NCPAP vs. N-BiPAP	2	24	353	32.0
Secondary outcomes – NEC				
NHFOV vs. NIPPV	2	140	10	20.2
NHFOV vs. NCPAP	1	80	5	10.6
NHFOV vs. N-BiPAP	1	65	2	7.2
NIPPV vs. NCPAP	8	839	37	33.0
NCPAP vs. N-BiPAP	3	405	19	29.0
Secondary outcomes – Mortality rate				
NHFOV vs. NIPPV	1	60	3	19.9
NHFOV vs. N-BiPAP	1	65	2	15.5
NIPPV vs. NCPAP	9	948	98	34.5
NCPAP vs. N-BiPAP	1	217	3	30.2

**Table 3:** League tables.

Primary outcome–Rate of reintubation within 72 h after extubation			
NHFOV	1.76 (1, 3.02)	3.71 (2.13, 6.87)	1.8 (0.88, 3.94)
0.57 (0.33, 1)*	NIPPV	2.11 (1.45, 3.32)	1.03 (0.54, 2.11)
0.27 (0.15, 0.47)*	0.47 (0.3, 0.69)*	NCPAP	0.49 (0.27, 0.88)
0.55 (0.25, 1.14)	0.97 (0.47, 1.86)	2.05 (1.14, 3.75)*	N-BiPAP
Primary outcome - PCO2			
NHFOV	24.51 (1.56, 409.8)	125.12 (5.42, 2,513.17)	21.05 (0.47, 1,122.74)
0.04 (0, 0.64)*	NIPPV	5.15 (0.24, 82.1)	0.86 (0.02, 47.34)
0.01 (0, 0.18)*	0.19 (0.01, 4.14)	NCPAP	0.17 (0.01, 6.47)
0.05 (0, 2.12)	1.16 (0.02, 52.24)	5.89 (0.15, 149.95)	N-BiPAP
Secondary outcomes - BPD			
NHFOV	1.06 (0.66, 1.74)	1.67 (1.03, 3.04)	1.21 (0.44, 3.43)
0.95 (0.57, 1.51)	NIPPV	1.58 (1.11, 2.44)	1.14 (0.43, 3.04)
0.6 (0.33, 0.97)*	0.63 (0.41, 0.9)*	NCPAP	0.72 (0.29, 1.72)
0.83 (0.29, 2.29)	0.87 (0.33, 2.31)	1.39 (0.58, 3.44)	N-BiPAP
Secondary outcomes - Nasal injuries			
NHFOV	1.17 (0.61, 2.21)		1.17 (0.57, 2.41)
0.86 (0.45, 1.64)	NIPPV		1.01 (0.61, 1.63)
0.86 (0.41, 1.77)	0.99 (0.61, 1.64)		NCPAP
Secondary outcomes - Air leak			
NHFOV	1.22 (0.39, 4.01)	2.53 (0.75, 10.33)	0.9 (0.08, 7.71)
0.82 (0.25, 2.56)	NIPPV	2.08 (0.89, 5.82)	0.75 (0.06, 6.92)
0.4 (0.1, 1.33)	0.48 (0.17, 1.13)	NCPAP	0.36 (0.02, 3.3)
1.11 (0.13, 12.63)	1.34 (0.14, 18.17)	2.81 (0.3, 43.07)	N-BiPAP
Secondary outcomes - IVH or PVL			
NHFOV	0.71 (0.4, 1.26)	0.83 (0.47, 1.47)	1.02 (0.49, 2.27)
1.41 (0.8, 2.53)	NIPPV	1.18 (0.84, 1.66)	1.46 (0.77, 2.83)
1.2 (0.68, 2.11)	0.85 (0.6, 1.19)	NCPAP	1.24 (0.71, 2.26)
0.98 (0.44, 2.04)	0.68 (0.35, 1.3)	0.81 (0.44, 1.41)	N-BiPAP
Secondary outcomes - ROP			
NHFOV	0.96 (0.32, 2.96)	1.18 (0.41, 3.43)	0.83 (0.19, 3.58)
1.04 (0.34, 3.11)	NIPPV	1.23 (0.75, 2)	0.87 (0.27, 2.61)
0.85 (0.29, 2.43)	0.81 (0.5, 1.33)	NCPAP	0.71 (0.25, 1.94)
1.2 (0.28, 5.31)	1.15 (0.38, 3.71)	1.41 (0.52, 4.06)	N-BiPAP
Secondary outcomes – NEC			
NHFOV	0.91 (0.27, 3.21)	1.23 (0.36, 4.52)	2.39 (0.52, 11.66)
1.1 (0.31, 3.64)	NIPPV	1.33 (0.65, 2.84)	2.62 (0.74, 9.56)
0.81 (0.22, 2.78)	0.75 (0.35, 1.54)	NCPAP	1.96 (0.68, 5.74)
0.42 (0.09, 1.92)	0.38 (0.1, 1.36)	0.51 (0.17, 1.47)	N-BiPAP
Secondary outcomes – Mortality rate			
NHFOV	1.98 (0.26, 20.02)	3.47 (0.44, 35.05)	1.29 (0.15, 13.21)
0.5 (0.05, 3.92)	NIPPV	1.75 (1.08, 2.9)	0.65 (0.13, 2.71)
0.29 (0.03, 2.28)	0.57 (0.35, 0.92)*	NCPAP	0.37 (0.08, 1.43)
0.77 (0.08, 6.69)	1.54 (0.37, 7.68)	2.69 (0.7, 12.68)	N-BiPAP

The data in the cells represent the MD and 95% CI values of the efficacy between the interventions in the corresponding columns and the rows of ten interventions. When the 95% *CI* includes the value 1, it indicates that the results are not statistically significant; conversely, if the 95% *CI* does not include 1, the results are considered statistically significant. When the MD is less than 1, it suggests that the interventions in the corresponding columns are superior to those in the rows, and the opposite indicates that the interventions in the rows are superior to those in the columns.

* Represents *P* < 0.05.

#### 3.4.2 PCO2 level within 24 h after changing to noninvasive assisted ventilation

A total of 12 studies (10, 12–14, 16, 19–21, 24, 29, 31, 32) involving 898 newborns were analyzed, demonstrating closed loops between all interventions as depicted in the network diagram (Figure 3). A significant portion of the data, representing 25.8%, was derived from comparisons between NHFOV and NIPPV (Table 2; Supplementary Material). A local inconsistency test using node splitting indicated *P* > 0.05, and the data were analyzed using a consistency model. The PSRF for all three outcome indicators converged to 1, suggesting good convergence (Supplementary Material). Heterogeneity was assessed separately for original studies with two or more contributions. Significant heterogeneity was observed, which might relate to variations in the timing of blood gas analysis and the types of diseases among the studies. The results of the network meta-analysis demonstrated that NHFOV was more effective than both NIPPV and NCPAP in facilitating carbon dioxide removal (both *P* < 0.05). However, no significant difference was observed between NIPPV and NCPAP (*P* > 0.05). No significant differences were found among the other three modes of respiratory support, excluding NHFOV (all *P* > 0.05) (Figure 4; Table 3). The SUCRA values for the four ventilation modes were 0.98, 0.45, 0.09, and 0.48, respectively, ranking the effectiveness in removing carbon dioxide post-extubation from highest to lowest as NHFOV>N-BiPAP>NIPPV>NCPAP. This highlights NHFOV's superiority in carbon dioxide removal compared to other ventilation strategies (Figure 5).

### 3.5 Secondary outcomes

#### 3.5.1 BPD

A total of 21 studies (10, 11, 13–18, 20–30) involving 2,163 children were analyzed. The results of the network meta-analysis indicated that NHFOV and NIPPV significantly reduced the odds of BPD compared to NCPAP, while no statistically significant difference was observed with N-BiPAP (*P* > 0.05). No significant differences were found among the other modes of respiratory support (all *P* > 0.05) (Figure 4; Table 3). The SUCRA values for NHFOV, NIPPV, NCPAP, and N-BiPAP were 0.73, 0.67, 0.08, and 0.50, respectively, suggesting a descending efficacy in reducing BPD from NHFOV to N-BiPAP to NIPPV to NCPAP (Figure 5).

#### 3.5.2 Nasal injuries

A review of seven studies (10, 11, 16, 21, 28, 31, 32) involving 621 children showed no statistically significant differences between the interventions (*P* > 0.05) (Figure 4; Table 3). The SUCRA values for NHFOV, NIPPV, NCPAP, and N-BiPAP were 0.68, 0.41, and 0.41, respectively (Figure 5).

#### 3.5.3 Air leakage

A total of 11 studies (11, 12, 14, 16, 19, 21, 22, 24, 28, 31, 32) involving 1,161 children were analyzed. The results of the network meta-analysis indicated no statistically significant differences between the interventions (*P* > 0.05) (Figure 4; Table 3). The SUCRA values for NIPPV, NCPAP, and N-BiPAP were 0.68, 0.57, 0.09 and 0.65, respectively (Figure 5).

#### 3.5.4 IVH or PVL

A total of 21 studies (10–17, 19–27, 29–32) including 2,145 children were reviewed. The network meta-analysis revealed no statistically significant differences between the interventions (*P* > 0.05) (Figure 4; Table 3). The SUCRA values for NHFOV, NIPPV, NCPAP, and N-BiPAP were 0.30, 0.87, 0.56, and 0.27, respectively (Figure 5).

#### 3.5.5 ROP, NEC

Network meta-analysis revealed no significant differences between the interventions (*P* > 0.05) (Figure 4; Table 3), and the ordination diagram is depicted in Figure 5.

#### 3.5.6 Mortality rate

The results of the network meta-analysis showed that NIPPV significantly reduced mortality compared to NCPAP, with no significant differences found between the other modes of respiratory support (all *P* > 0.05) (Figure 4; Table 3).

#### 3.5.7 Quality of evidence

The overall confidence level of the network meta-analysis (NMA) effect estimate for reintubation within 72 h was moderate for nasal high-flow oxygen ventilation (NHFOV) compared with noninvasive positive pressure ventilation (NIPPV) and nasal continuous positive airway pressure (NCPAP). The confidence level for CO2 retention within 24 h was rated as moderate for NHFOV vs. NIPPV, and low to very low when directly compared to other interventions. The quality of evidence for all other secondary outcomes ranged from very low to moderate across comparisons. Refer to Table 4 for the quality of evidence for all outcome comparisons.

**Table 4:** Quality of evidence/GRADE.

Primary outcome–Rate of reintubation within 72 h after extubation				
Comparison	Nature of the evidence	Confidence	Downgrading due to	Risk ratio
NHFOV vs. NIPPV	Mixed	Moderate	a	0.57 (0.33, 1)*
NHFOV vs. NCPAP	Mixed	Moderate	a	0.27 (0.15, 0.47)*
NHFOV vs. N-BiPAP	Mixed	Low	ad	0.55 (0.25, 1.14)
NIPPV vs. NCPAP	Mixed	Moderate	a	0.47 (0.3, 0.69)*
NIPPV vs. N-BiPAP	Mixed	Low	ad	0.97 (0.47, 1.86)
NCPAP vs. N-BiPAP	Mixed	Low	ab	2.05 (1.14, 3.75)*
Primary outcome - PCO2 level within 24 h after changing to noninvasive assisted ventilation				
NHFOV vs. NIPPV	Mixed	Moderate	a	0.04 (0, 0.64)*
NHFOV vs. NCPAP	Mixed	Low	ab	0.01 (0, 0.18)*
NHFOV vs. N-BiPAP	Mixed	Very low	abd	0.05 (0, 2.12)
NIPPV vs. NCPAP	Mixed	Very low	abd	0.19 (0.01, 4.14)
NIPPV vs. N-BiPAP	Mixed	Low	ad	1.16 (0.02, 52.24)
NCPAP vs. N-BiPAP	Mixed	Very low	abd	5.89 (0.15, 149.95)
Secondary outcomes - BPD				
NHFOV vs. NIPPV	Mixed	Low	ad	0.95 (0.57, 1.51)
NHFOV vs. NCPAP	Mixed	Very low	abd	0.6 (0.33, 0.97)*
NHFOV vs. N-BiPAP	Mixed	Low	ad	0.83 (0.29, 2.29)
NIPPV vs. NCPAP	Mixed	Moderate	a	0.63 (0.41, 0.9)*
NIPPV vs. N-BiPAP	Indirect	Very low	acd	0.87 (0.33, 2.31)
NCPAP vs. N-BiPAP	Mixed	Low	ad	1.39 (0.58, 3.44)
Secondary outcomes - Nasal injuries				
NHFOV vs. NIPPV	Mixed	Low	ad	0.86 (0.45, 1.64)
NHFOV vs. NCPAP	Mixed	Low	ad	0.86 (0.41, 1.77)
NIPPV vs. NCPAP	Mixed	Low	ad	0.99 (0.61, 1.64)
Secondary outcomes - Air leak				
NHFOV vs. NIPPV	Mixed	Low	ad	0.82 (0.25, 2.56)
NHFOV vs. NCPAP	Mixed	Low	ad	0.4 (0.1, 1.33)
NHFOV vs. N-BiPAP	Mixed	Very low	abd	1.11 (0.13, 12.63)
NIPPV vs. NCPAP	Mixed	Low	ad	0.48 (0.17, 1.13)
NIPPV vs. N-BiPAP	Mixed	Low	ad	1.34 (0.14, 18.17)
NCPAP vs. N-BiPAP	Mixed	Very low	abd	2.81 (0.3, 43.07)
Secondary outcomes - IVH or PVL				
NHFOV vs. NIPPV	Mixed	Low	ad	1.41 (0.8, 2.53)
NHFOV vs. NCPAP	Mixed	Low	ad	1.2 (0.68, 2.11)
NHFOV vs. N-BiPAP	Mixed	Low	ad	0.98 (0.44, 2.04)
NIPPV vs. NCPAP	Mixed	Moderate	a	0.85 (0.6, 1.19)
NIPPV vs. N-BiPAP	Mixed	Low	ad	0.68 (0.35, 1.3)
NCPAP vs. N-BiPAP	Mixed	Low	ad	0.81 (0.44, 1.41)
Secondary outcomes - ROP				
NHFOV vs. NIPPV	Mixed	Low	ad	1.04 (0.34, 3.11)
NHFOV vs. NCPAP	Mixed	Low	ad	0.85 (0.29, 2.43)
NHFOV vs. N-BiPAP	Indirect	Low	ac	1.2 (0.28, 5.31)
NIPPV vs. NCPAP	Mixed	Low	ad	0.81 (0.5, 1.33)
NIPPV vs. N-BiPAP	Indirect	Low	ac	1.15 (0.38, 3.71)
NCPAP vs. N-BiPAP	Mixed	Moderate	a	1.41 (0.52, 4.06)
Secondary outcomes – NEC				
NHFOV vs. NIPPV	Mixed	Low	ad	1.1 (0.31, 3.64)
NHFOV vs. NCPAP	Mixed	Low	ad	0.81 (0.22, 2.78)
NHFOV vs. N-BiPAP	Mixed	Low	ad	0.42 (0.09, 1.92)
NIPPV vs. NCPAP	Mixed	Low	ad	0.75 (0.35, 1.54)
NCPAP vs. N-BiPAP	Mixed	Low	ad	0.51 (0.17, 1.47)
Secondary outcomes – Mortality rate				
NHFOV vs. NIPPV	Mixed	Low	ad	0.5 (0.05, 3.92)
NHFOV vs. NCPAP	Indirect	Very low	acd	0.81 (0.22, 2.78)
NHFOV vs. N-BiPAP	Mixed	Low	ad	0.77 (0.08, 6.69)
NIPPV vs. NCPAP	Mixed	Moderate	a	0.57 (0.35, 0.92)*
NCPAP vs. N-BiPAP	Mixed	Low	ad	2.69 (0.7, 12.68)

GRADE ranking the quality of evidence.

High quality—very confident that the true effect lies close to that of the estimate of the effect.

Moderate quality—moderately confident in the effect estimate: the true effect is likely to be close to the estimate of the effect, but there is a possibility.

that it is substantially different.

Low quality—confidence in the effect estimate is limited: the true effect may be substantially different from the estimate of the effect.

Very low quality—very little confidence in the effect estimate: the true effect is likely to be substantially different from the estimate of effect.

BiPAP, bilevel CPAP; bmp, beats per minute; CPAP, continuous positive airway pressure; HFNC, humidified high flow cannula; NIPPV, noninvasive positive pressure ventilation.

^a^Limitations (risk of bias).

^b^Inconsistency.

^c^Indirectness.

^d^Imprecision.

^e^Iother consideration.

*Indicates significant.

#### 3.5.8 Publication bias test

**Figure 6:**
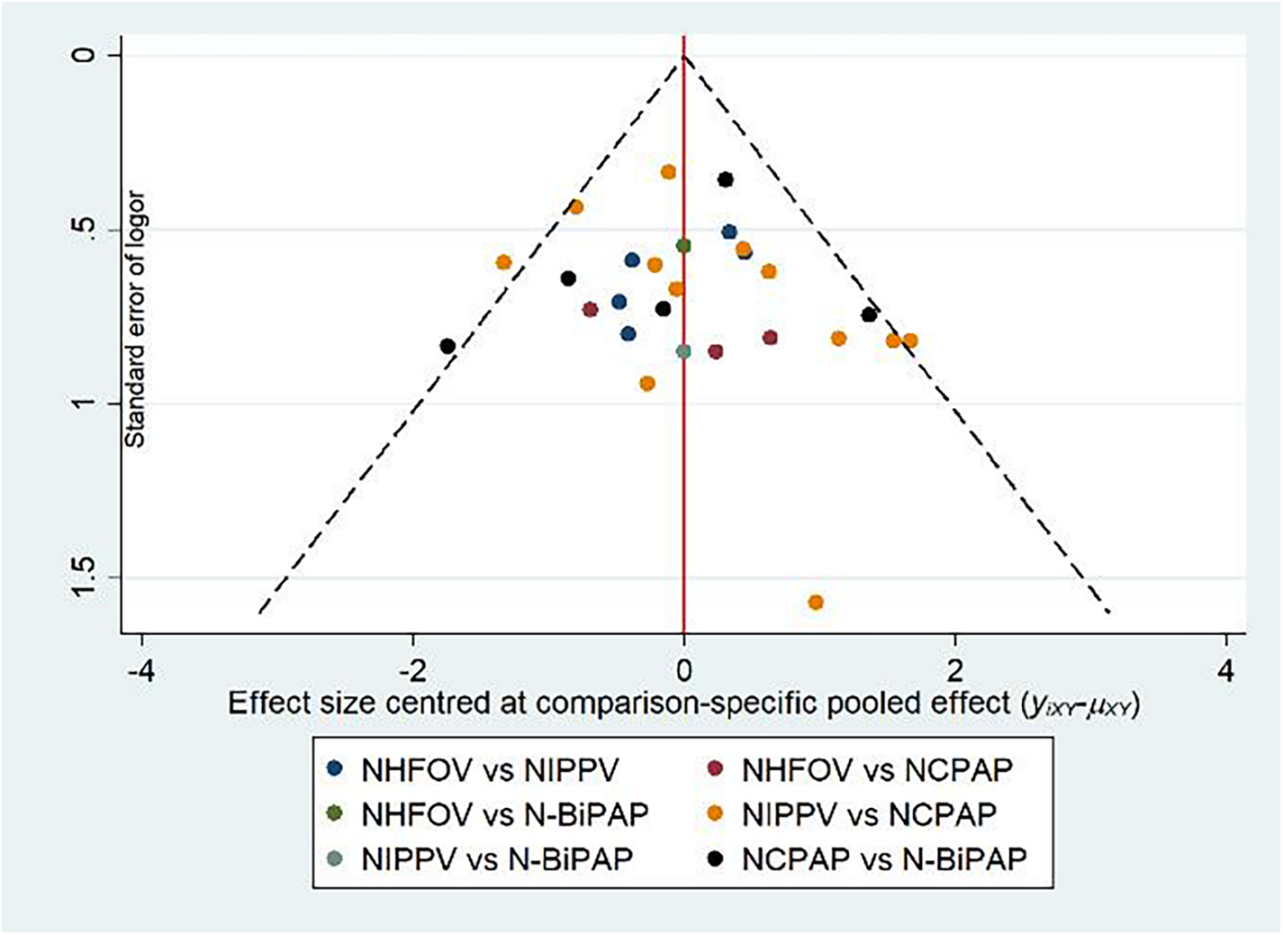
SUCRA plots for the outcomes.

A corrected one-comparison funnel plot focusing on 72 h reintubation as the primary outcome showed a concentrated scatter in the upper middle with good symmetry, indicating no significant publication bias (Figure 6).

## 4 Discussion

Currently, mechanical ventilation has a primary treatment modality in NRDS due to its irreplaceable role (35). Critically ill neonates exhibit low resistance, and prolonged mechanical ventilation may increase the risk of ventilator-associated complications such as lung injury and infection (36). Consequently, identifying a safer and more effective noninvasive respiratory support methods post-extubation was crucial. This study evaluated various NRS modalities, focusing on outcomes like reintubation and carbon dioxide retention following extubation in 2,331 neonates across 23 studies.

The results of this NMA study indicated that NHFOV is potentially the most effective NRS mode for post-extubation respiratory support, compared to NIPPV, NCPAP, and N-BiPAP. From biomechanical and physiological perspectives (37), the high-frequency oscillations provided by NHFOV improve lung ventilation homogeneity, minimize ventilation/blood flow mismatch, and may reduce lung injury by stabilizing the ventilation pressure gradient (38). This study supported these findings, showing that NHFOV significantly reduced reintubation risks, enhanced carbon dioxide removal, and lowered the incidence of BPD. Czernik et al. (39) and Seth et al. (14) reported that NHFOV effectively addressed extubation challenges and outperformed NIPPV in reducing CO2 retention. However, Ramaswamy and Wu et al. (40, 41) conclusions are in contrast to this study, where they showed that NIPPV performed best in reducing the need for mechanical ventilation, while high-frequency oscillatory ventilation (nHFOV) was the least effective. This may be due to the fact that the effectiveness of nHFOV has been more widely validated and optimized in recent years with the continuous advancement of nHFOV equipment and techniques, especially in premature infants with respiratory distress syndrome (RDS). Moreover, that study focused on the initial treatment of noninvasive breathing in NRDS, whereas this study focused on the efficacy and safety of noninvasive respiratory support after extubation, and our results added to the existing literature.

The results of this NMA analysis indicated that NIPPV was also employed as a mode of respiratory support post-extubation. Although less effective than NHFOV in preventing reintubation within 72 h, NIPPV significantly reduced the reintubation rates among children with NRDS, demonstrating greater clinical efficacy than NCPAP and N-BiPAP. Ramanathan (42) reported that NIPPV, by incorporating intermittent positive pressure into NCPAP, provides stronger respiratory support and reduces reintubation rates.

In this study, N-BiPAP was shown to significantly decrease the risk of reintubation compared to NCPAP, a finding supported by Victor et al. (43) and consistent with existing literature (32, 44, 45). N-BiPAP may enhance respiratory outcomes by reducing thoracoabdominal asynchrony and airway resistance, dilating the airways with peak inspiratory pressure (PIP), increasing tidal volume and minute ventilation, expanding the collapsed trachea, and enhancing functional residual capacity. Notably, the PaCO2 levels post-extubation were lower with N-BiPAP than with NCPAP, attributed to the effective bi-level airway pressure support of N-BiPAP which stimulates breathing and provides robust respiratory support. However, no significant differences were observed in CO2 clearance compared to other interventions; this could be due to the higher frequency of re-tracheal intubation within 24 h post-extubation in the sickest children in the NCPAP group, which improved respiratory support, making the differences statistically insignificant when compared with the N-BiPAP group.

Although this study indicated that NCPAP may not be as effective as newer noninvasive respiratory support modalities in reducing the risk of reintubation and enhancing carbon dioxide elimination, its significance should not be overlooked. NCPAP, a traditional method in NRDS management, boasts extensive clinical application and research, effectively maintaining alveolar stability by providing continuous positive airflow, which prevents alveolar collapse and enhances lung ventilation (46). Thus, based on current clinical research data, it is recommended that the appropriate NRS mode be selected according to the specific clinical condition of the neonate and the respiratory support required.

Regarding safety, the results of this NMA analysis demonstrated that the NHFOV group significantly lowered the incidence of BPD compared to both the NIPPV and NCPAP groups, aligning with findings by Null (47). However, no significant differences were observed among the intervention groups concerning complications such as air leakage, nasal injury, PVL, IVH, and ROP, suggesting comparable safety across these groups. Consequently, all four noninvasive ventilation strategies were promotable and applicable in clinical settings as effective and safe noninvasive ventilation modes.

In recent years, the INSURE technique has gained increased use in clinical practice, aimed at minimizing the potential harm caused by mechanical ventilation (MV) in neonates. This method markedly reduces the duration of intubation by utilizing a therapeutic approach that involves endotracheal intubation, surfactant administration, and subsequent non-invasive ventilation (NIV) post-extubation. Frequently utilized NIV modes include nasal continuous positive airway pressure (NCPAP), and more recently, nasal intermittent positive pressure ventilation (NIPPV) has also gained widespread adoption in clinical settings. It is also noteworthy that the Laryngeal Mask Airway (LMA) can serve as an effective alternative when mechanical ventilation is not feasible. Studies have demonstrated that LMA not only provides airway support but also facilitates the administration of surfactant, thereby enhancing neonatal lung function without requiring endotracheal intubation. De Bernardo et al. highlighted that delivering surfactant via LMA, while simultaneously monitoring oxygen saturation, is a minimally invasive and effective intervention that facilitates a smoother transition from invasive to noninvasive mechanical ventilation. This approach helps to mitigate the complications associated with invasive mechanical ventilation and enables early initiation of noninvasive support. As a result, LMA may be considered a valuable bridging technique in subsequent treatment strategies, particularly for preterm infants who are not suitable candidates for tracheal intubation (48, 49).

### 4.1 Shortcomings and limitations of this meta-analysis

Limitations of this NMA included: (1) Variability in the number of studies analyzed for different noninvasive ventilation strategies, with some having fewer interventions and smaller sample sizes. (2) A limited number of NRS modalities were included in this analysis; notably, modalities such as humidified high-flow nasal catheter ventilation (HFNC) were excluded. (3) Inconsistencies were observed in one secondary outcome (air leak). (4) Most of the data in this study were derived from Asian populations and thus may have some limitations in terms of generalisability across ethnicities and regions. Future research should expand the geographic and ethnic diversity of the study sample to more fully validate the generalisability and applicability of the findings. (5) The literature included in this study was not clearly harmonised in terms of diagnostic criteria for RDS.

## 5 Conclusions

Current evidence indicates that NHFOV significantly reduced the need for mechanical ventilation in NRDS compared to other ventilation modes, enhanced the success rate of extubation, and decreased the incidence of BPD. However, it had not been conclusively demonstrated that each intervention significantly lowered the incidence of complications such as nasal injury, air leakage, IVH, ROP, and NEC. Furthermore, the efficacy of each noninvasive ventilation strategy post-extubation in NRDS requires further investigation through large-scale clinical multicenter RCTs.

## Data Availability

The datasets presented in this study can be found in online repositories. The names of the repository/repositories and accession number(s) can be found in the article/Supplementary Material.
