# Supplementary material for: Efficacy and safety of different noninvasive ventilation strategies for postextubation respiratory support in Neonatal Respiratory Distress Syndrome: a systematic review and network meta-analysis
Source: Front Pediatr. 2024 Nov 15;12:1435518. doi: 10.3389/fped.2024.1435518 (PMC11607678; doi:10.3389/fped.2024.1435518)

Table S1 Search Strategy		
Search number	Query	Results
1	"High-Frequency Ventilation"[Mesh]	3,024
2	High-Frequency Ventilations[Title/Abstract] OR Ventilations, High-Frequency[Title/Abstract] OR Ventilation, High Frequency[Title/Abstract] OR Ventilation, High-Frequency[Title/Abstract] OR High-Frequency Ventilation High Frequency Ventilation[Title/Abstract] OR Ventilations, High Frequency[Title/Abstract] OR High-Frequency Oscillation Ventilation[Title/Abstract] OR High-Frequency Oscillation Ventilation[Title/Abstract] OR Oscillation Ventilation, High-Frequency[Title/Abstract] OR Oscillation Ventilations, High-Frequency[Title/Abstract] OR Ventilation, High-Frequency Oscillation[Title/Abstract] OR Ventilations, High-Frequency Oscillation[Title/Abstract] OR High-Frequency Oscillation Ventilation[Title/Abstract] OR High-Frequency Positive Pressure Ventilation[Title/Abstract] OR High-Frequency Positive Pressure Ventilation[Title/Abstract] OR Noninvasive high-frequency oscillatory ventilation[Title/Abstract] OR NHFOV[Title/Abstract]	5,027
3	"Continuous Positive Airway Pressure"[Mesh]	9,517
4	CPAP Ventilation[Title/Abstract] OR Ventilation, CPAP[Title/Abstract] OR Nasal Continuous Positive Airway Pressure[Title/Abstract] OR nCPAP Ventilation[Title/Abstract] OR Ventilation, nCPAP[Title/Abstract] OR Airway Pressure Release Ventilation[Title/Abstract] OR APRV Ventilation Mode[Title/Abstract] OR APRV Ventilation Modes[Title/Abstract] OR Ventilation Mode, APRV[Title/Abstract] OR Ventilation Modes, APRV[Title/Abstract] OR Biphasic Continuous Positive Airway Pressure[Title/Abstract] OR BiPAP Biphasic Positive Airway Pressure[Title/Abstract] OR BiPAP Bilevel Positive Airway Pressure[Title/Abstract] OR Biphasic Positive Airway Pressure[Title/Abstract] OR Bilevel Continuous Positive Airway Pressure[Title/Abstract] OR Bilevel Positive Airway Pressure[Title/Abstract] OR NCPAP[Title/Abstract]	3,867
5	"Intermittent Positive-Pressure Ventilation"[Mesh]	2,432
6	Intermittent Positive Pressure Ventilation[Title/Abstract] OR Positive-Pressure Ventilation, Intermittent[Title/Abstract] OR IPPV[Title/Abstract] OR Ventilation, Intermittent Positive-Pressure[Title/Abstract] OR Ventilation, Intermittent Positive Pressure[Title/Abstract] OR Inspiratory Positive-Pressure Ventilation[Title/Abstract] OR Inspiratory Positive Pressure Ventilation[Title/Abstract] OR Positive-Pressure Ventilation, Inspiratory[Title/Abstract] OR Ventilation, Inspiratory Positive-Pressure[Title/Abstract] OR Biphasic Intermittent Positive Airway Pressure[Title/Abstract] OR BIPAP Biphasic Intermittent Positive Airway Pressure[Title/Abstract] OR nasal intermittent positive pressure ventilation[Title/Abstract] OR interval positive pressure ventilation[Title/Abstract] OR NIPPV[Title/Abstract]	2,665
7	Biphasic Continuous Positive Airway Pressure[Title/Abstract] OR BiPAP Biphasic Positive Airway Pressure[Title/Abstract] OR BiPAP Bilevel Positive Airway Pressure[Title/Abstract] OR Biphasic Positive Airway Pressure[Title/Abstract] OR Bilevel Continuous Positive Airway Pressure[Title/Abstract] OR Bilevel Positive Airway Pressure[Title/Abstract] OR nasal biphasic positive airway pressure ventilation[Title/Abstract] OR nBiPAP[Title/Abstract]	789
8	#1 or #2 or #3 or #4 or #5 or #6 or #7	20,920
9	Infantile Respiratory Distress Syndrome[Title/Abstract] OR Neonatal Respiratory Distress Syndrome[Title/Abstract] OR Respiratory Distress Syndrome, Infant[Title/Abstract] OR Distress Syndrome, Respiratory[Title/Abstract] OR Distress Syndromes, Respiratory[Title/Abstract] OR Respiratory Distress	63,087

	Syndromes[Title/Abstract] OR Syndrome, Respiratory Distress[Title/Abstract] OR hyaline membrance disease of lung[Title/Abstract] OR Respiratory Distress Syndrome, Pediatric[Title/Abstract] OR Pediatric Respiratory Distress Syndrome	
10	Infants, Newborn[Title/Abstract] OR Newborn Infant[Title/Abstract] OR Newborn Infants[Title/Abstract] OR Newborns[Title/Abstract] OR Newborn[Title/Abstract] OR Neonate[Title/Abstract] OR Neonates[Title/Abstract] OR Infants, Premature[Title/Abstract] OR Premature Infant[Title/Abstract] OR Preterm Infants[Title/Abstract] OR Infant, Preterm[Title/Abstract] OR Infants, Preterm[Title/Abstract] OR Preterm Infant[Title/Abstract] OR Premature Infants[Title/Abstract] OR Neonatal Prematurity[Title/Abstract] OR Prematurity, Neonatal[Title/Abstract]	333,181
11	#9 or #10	4,942
12	#8 AND #11	384,169
13	randomized controlled trial[Publication Type] OR randomized[Title/Abstract] OR placebo[Title/Abstract]	1,061,795
14	#12 AND #13	1,041

Supplementary Figure 1: Network Contribution Graph

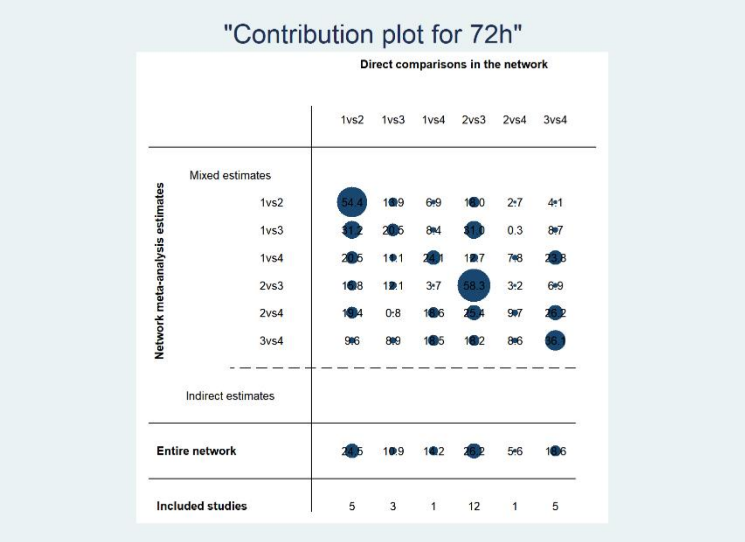

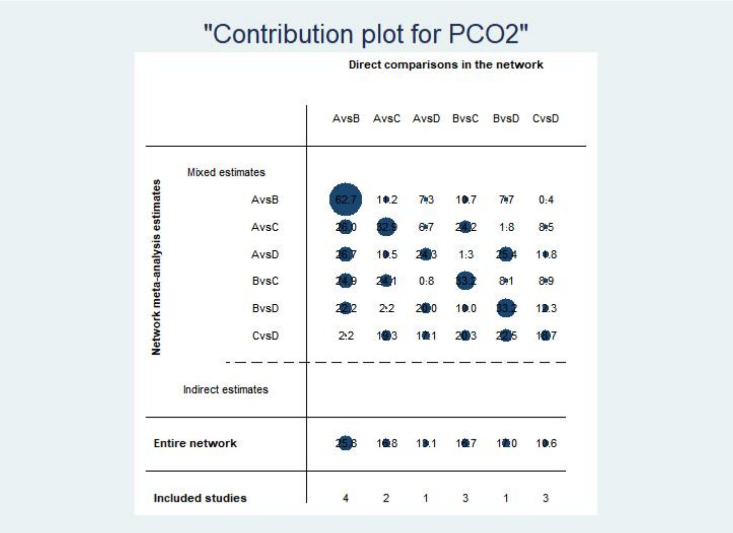

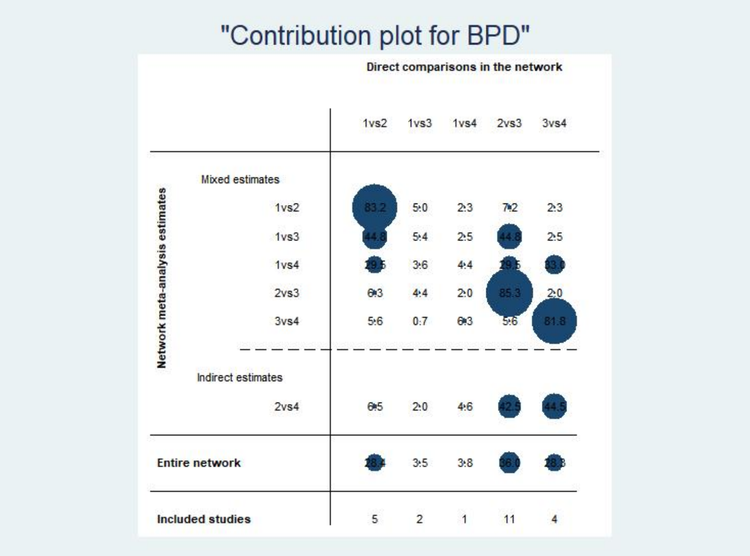

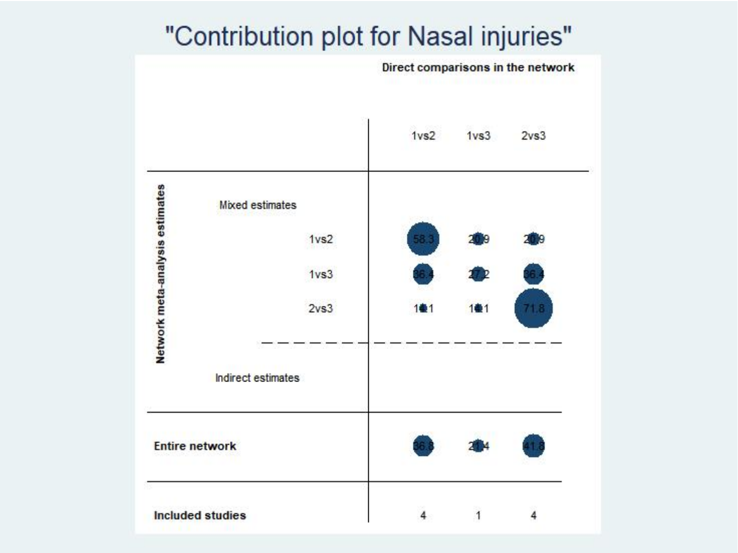

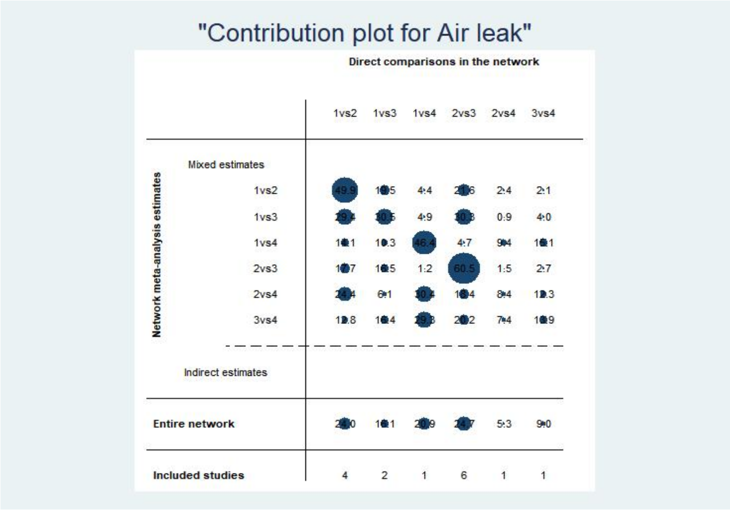
"Contribution plot for Air leak"
Direct comparisons in the network
1vs2 1vs3 1vs4 2vs3 2vs4 3vs4
Network meta-analysis estimates
Mixed estimates
1vs2
1vs3
1vs4
2vs3
2vs4
3vs4
Indirect estimates
Entire network
Included studies

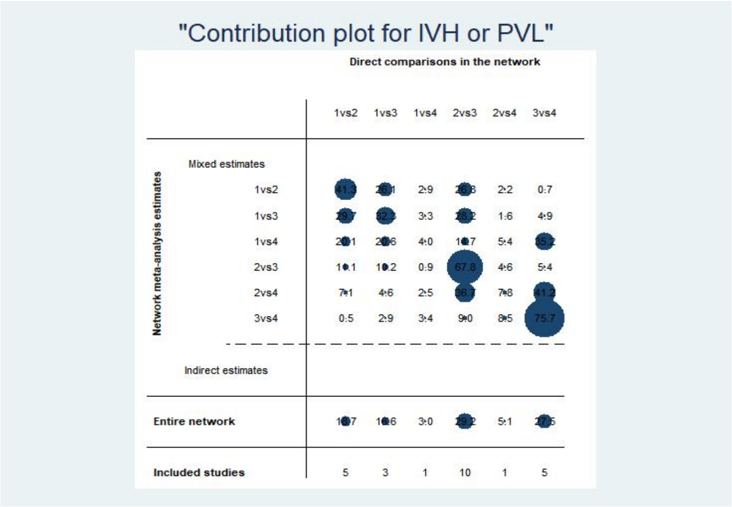
"Contribution plot for IVH or PVL"
Direct comparisons in the network
1vs2 1vs3 1vs4 2vs3 2vs4 3vs4
Network meta-analysis estimates
Mixed estimates
1vs2
1vs3
1vs4
2vs3
2vs4
3vs4
Indirect estimates
Entire network
Included studies

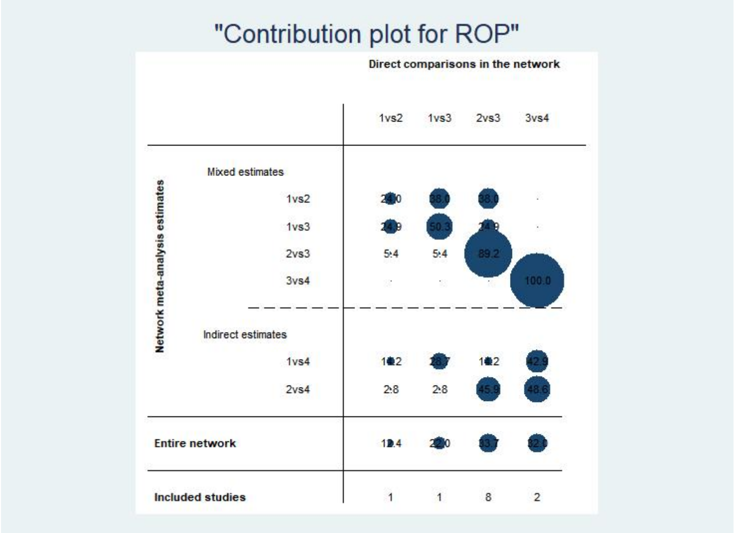
"Contribution plot for ROP"
Direct comparisons in the network
1vs2 1vs3 2vs3 3vs4
Network meta-analysis estimates
Mixed estimates
1vs2
1vs3
2vs3
3vs4
Indirect estimates
1vs4
2vs4
Entire network
Included studies

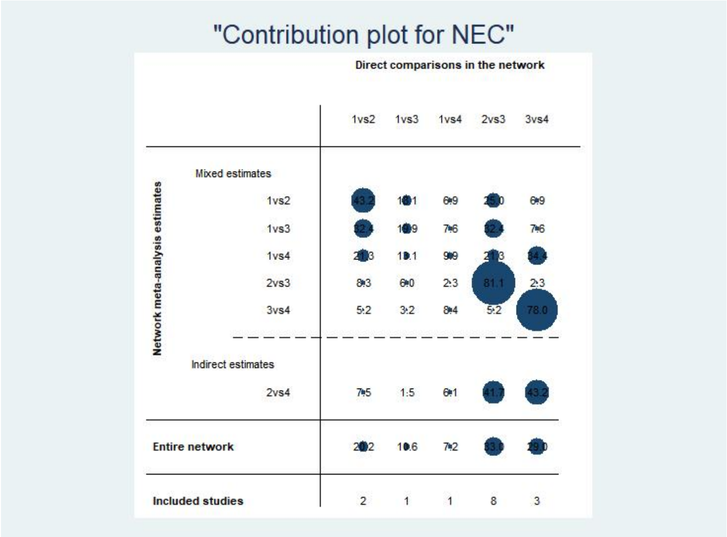
"Contribution plot for NEC"
Direct comparisons in the network
1vs2 1vs3 1vs4 2vs3 3vs4
Network meta-analysis estimates
Mixed estimates
1vs2
1vs3
1vs4
2vs3
3vs4
Indirect estimates
2vs4
Entire network
Included studies

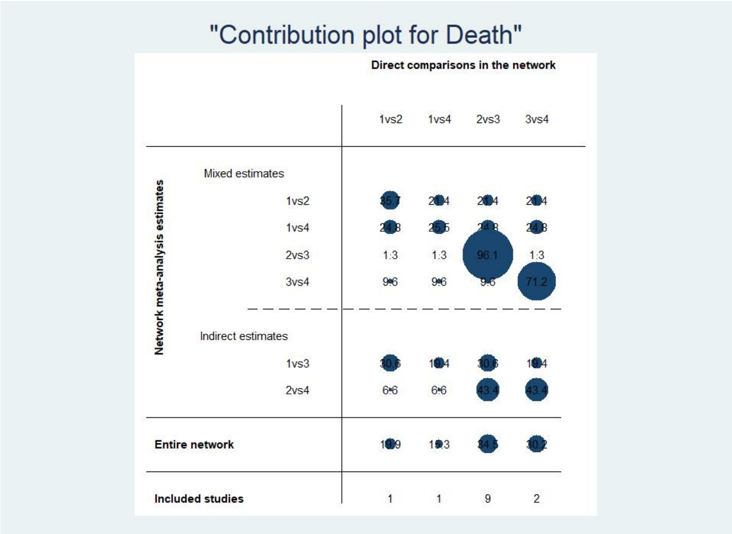
"Contribution plot for Death"
Direct comparisons in the network
1vs2 1vs4 2vs3 3vs4
Network meta-analysis estimates
Mixed estimates
1vs2
1vs4
2vs3
3vs4
Indirect estimates
1vs3
2vs4
Entire network
Included studies

Supplementary Figure 2: Inconsistency test

（A： NHFOV ; B:NIPPV ; C:NCPAP ; D:N-BiPAP ）

(1) 72h re-intubation

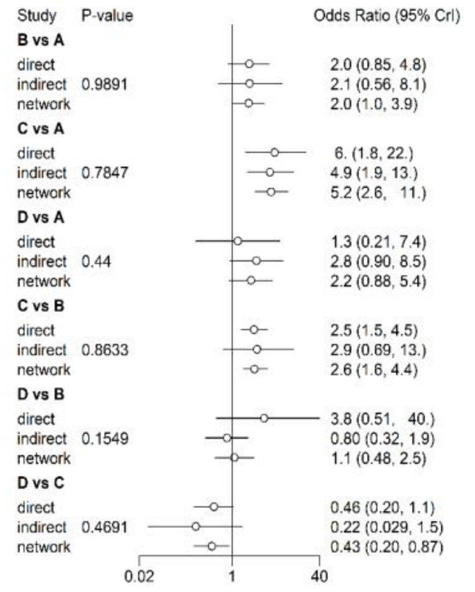

(2) PCO2

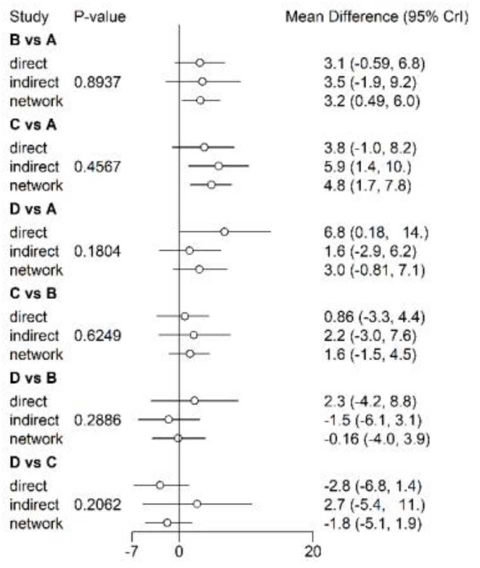

(3) BPD

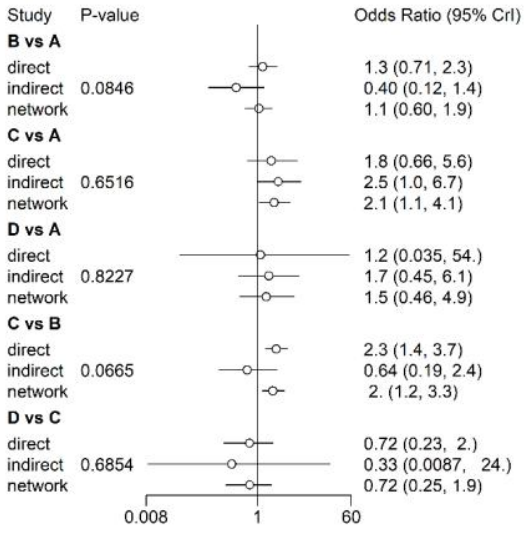

(4) Nasal injuries

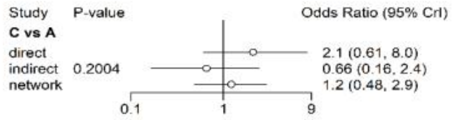

(5) Air leaks

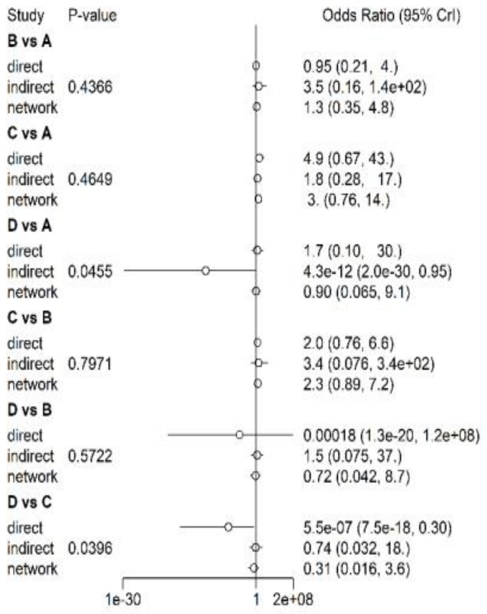

(6) IVH or PVL

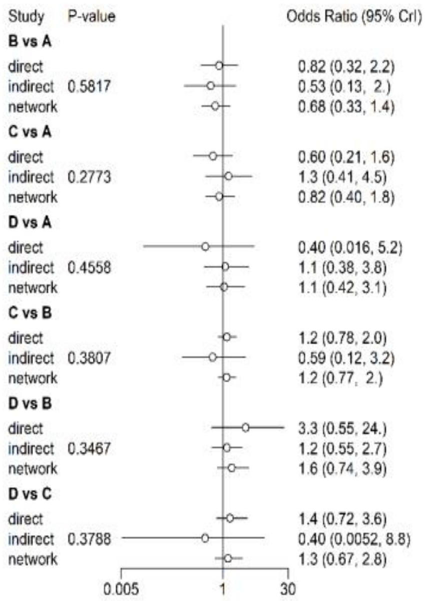

(7) ROP

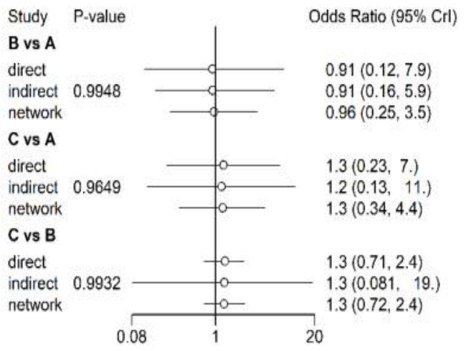

(8) NEC

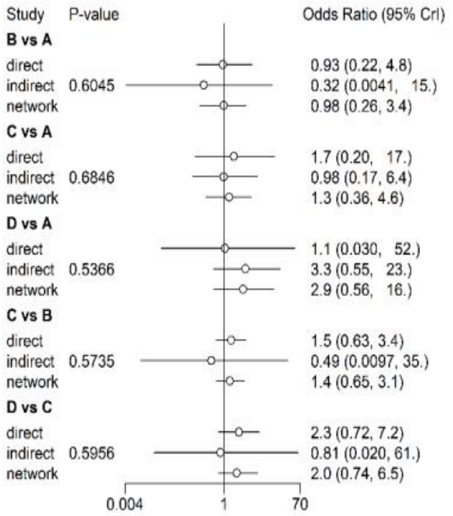

Supplementary Figure 3: Trajectory plot and density plot

（A：NHFOV ; B:NIPPV ; C:NCPAP ; D:N-BiPAP ）

(1) 72h re-intubation

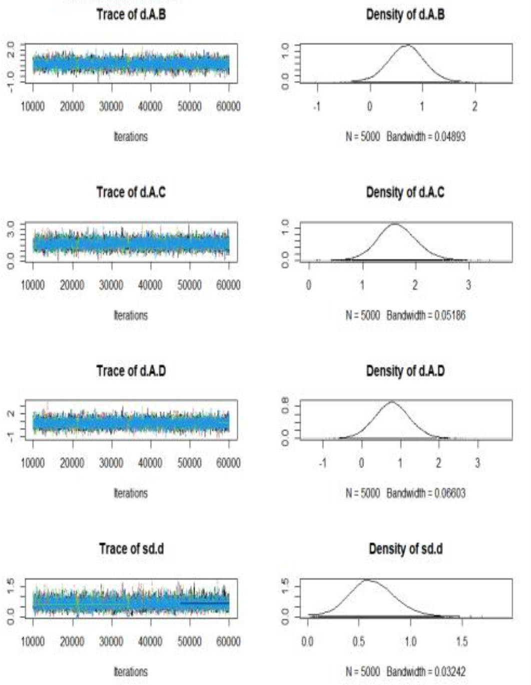

(2) PCO2

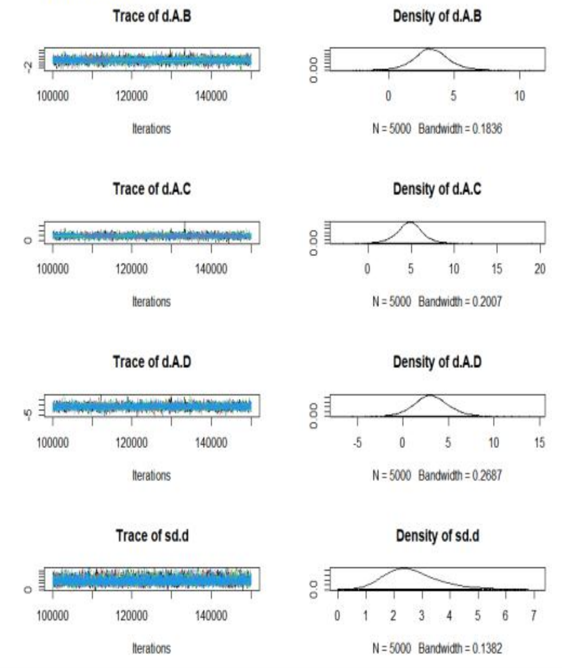

(3) BPD

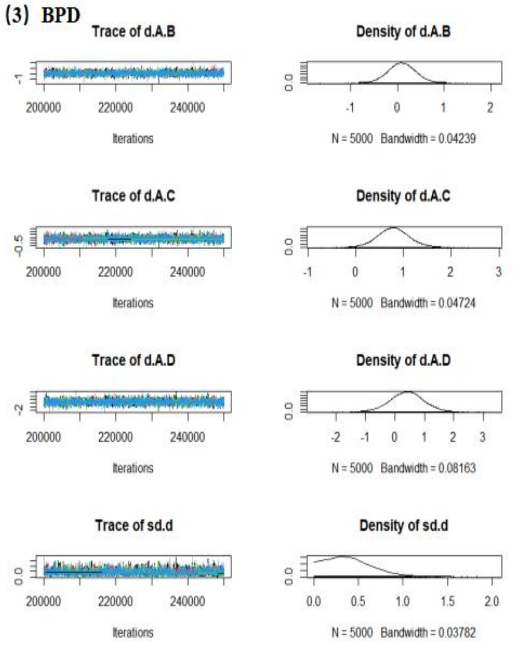

(4) Nasal injuries

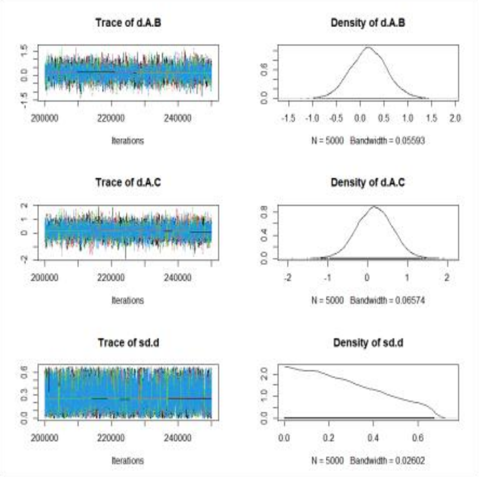

(5) Air leaks

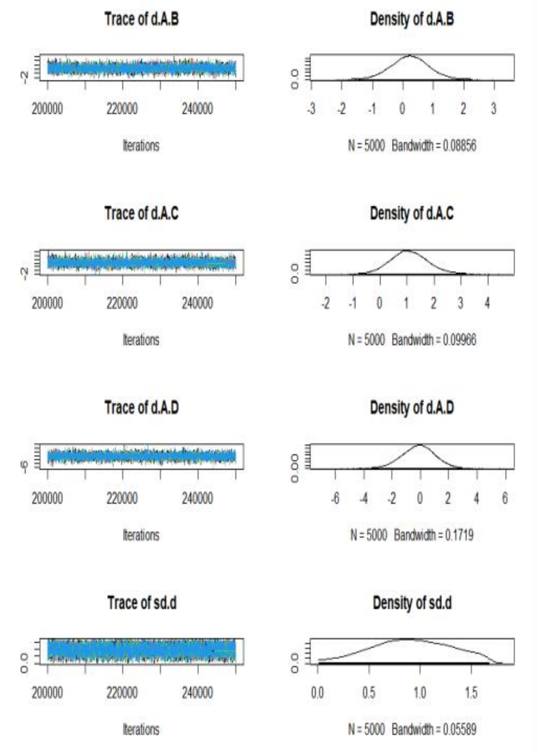

(6) IVH or PVL

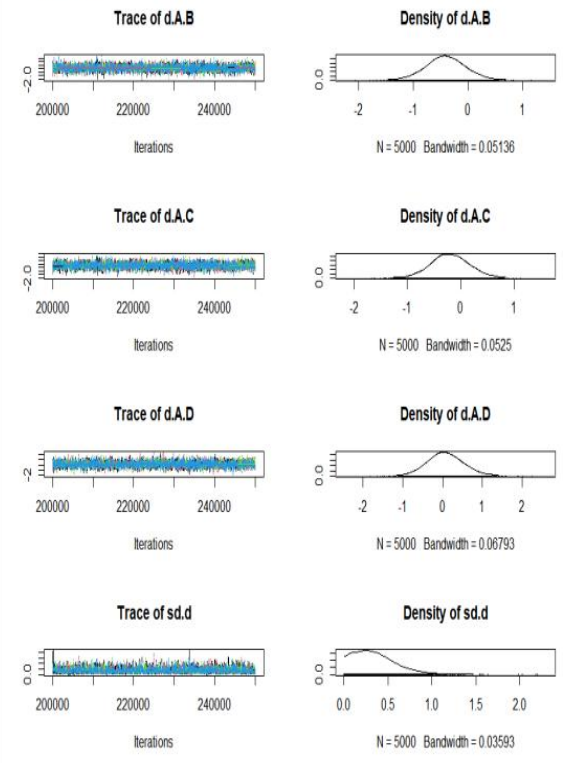

(7) ROP

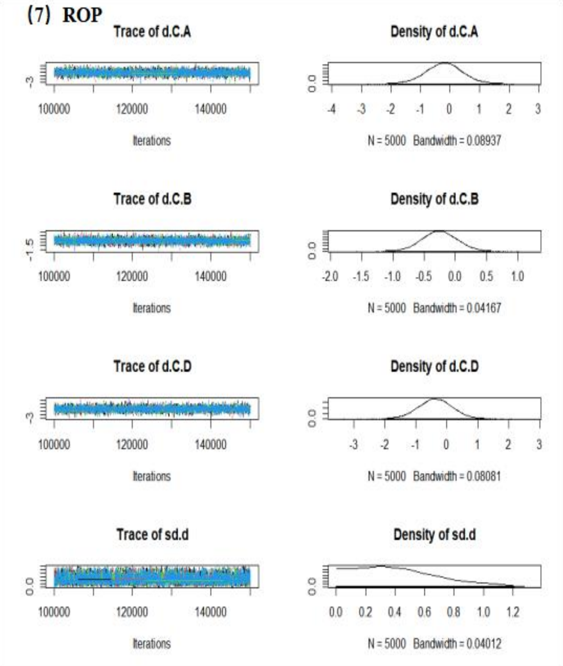

(8) NEC

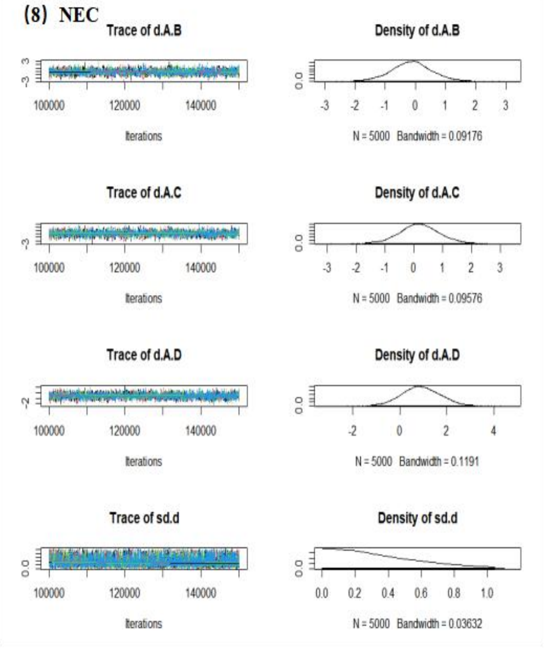

(9) Death

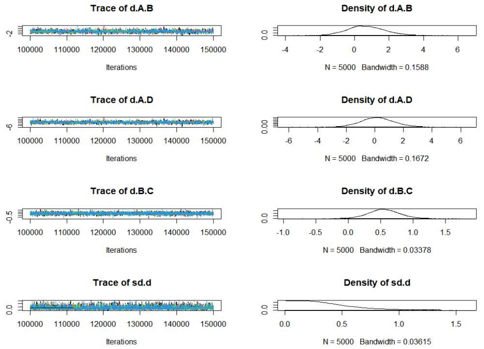

Supplementary Figure 4: Diagnostic convergence plot

（A：NHFOV ; B:NIPPV ; C:NCPAP ; D:N-BiPAP ）

(1) 72h re-intubation

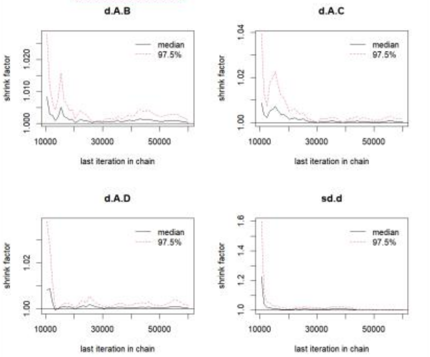

(2) PCO2

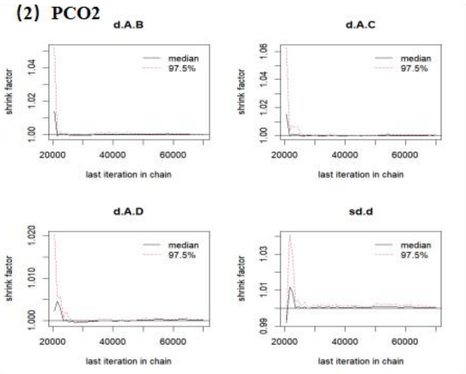

(3) BPD

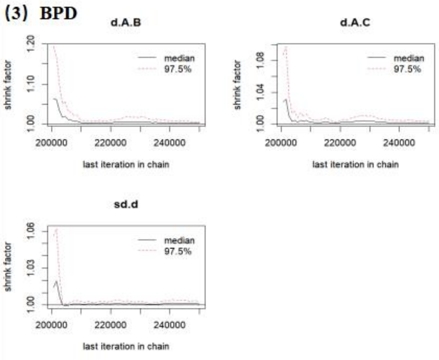

(4) Nasal injuries

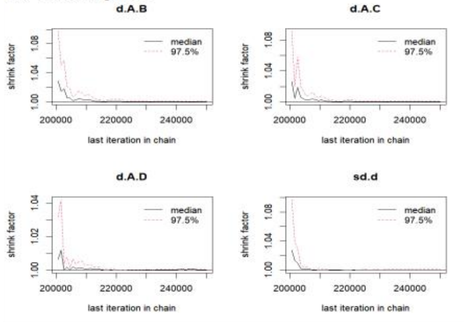

(5) Air leaks

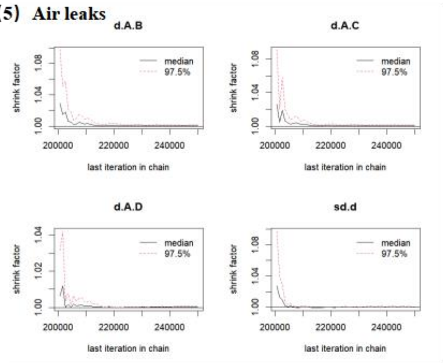

(6) IVH or PVL

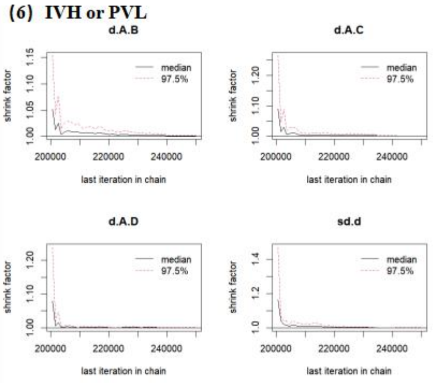

(7) ROP

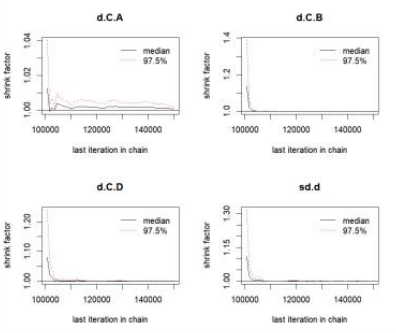

(8) NEC

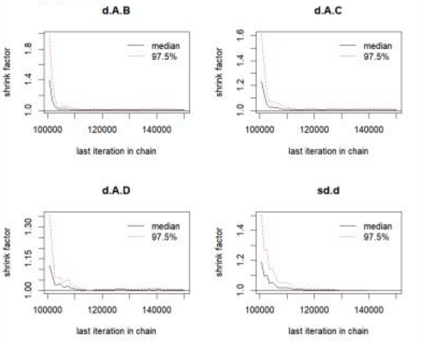

(9) Death

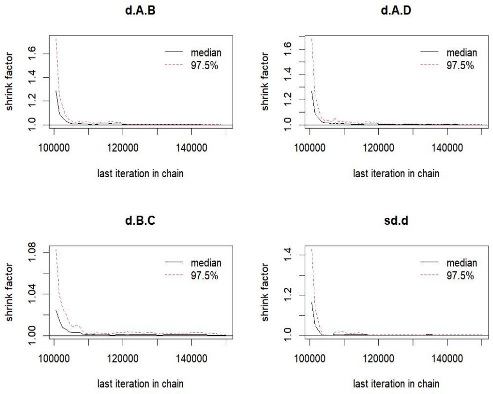

d.A.B
d.A.D
d.B.C
sd.d
median
97.5%
shrink factor
last iteration in chain
100000
120000
140000

Supplementary Figure 5: Heterogeneity test

(1) 72h re-intubation

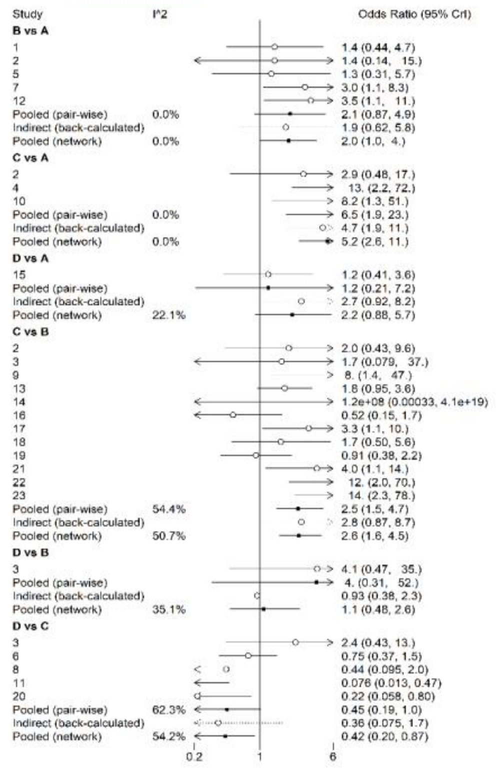

(2) PCO2

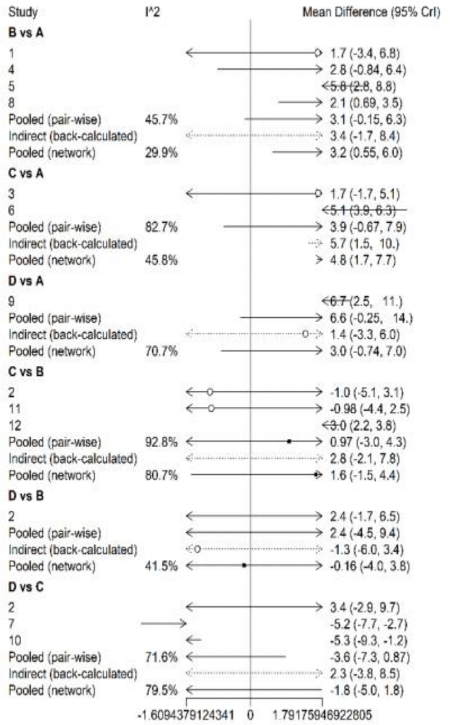

(3) BPD

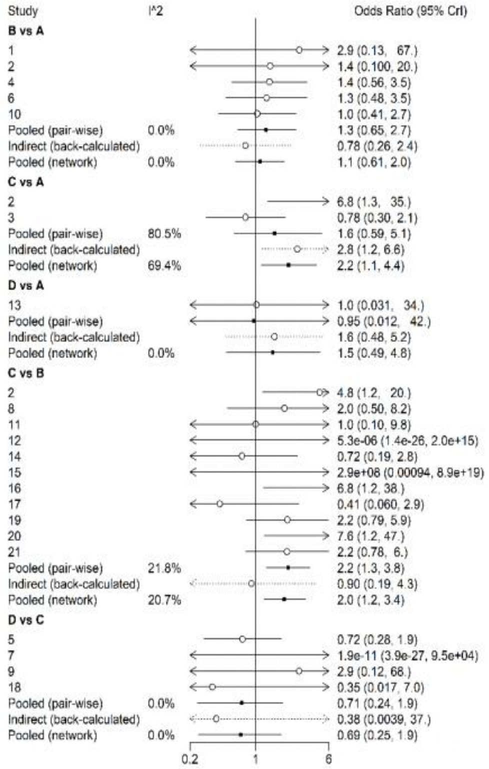

(3) Nasal injuries

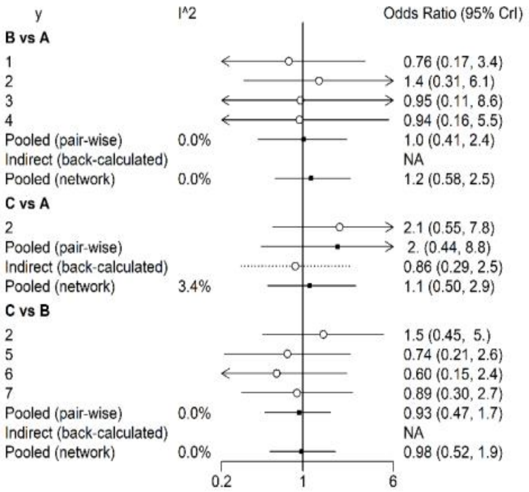

(5) Air leaks

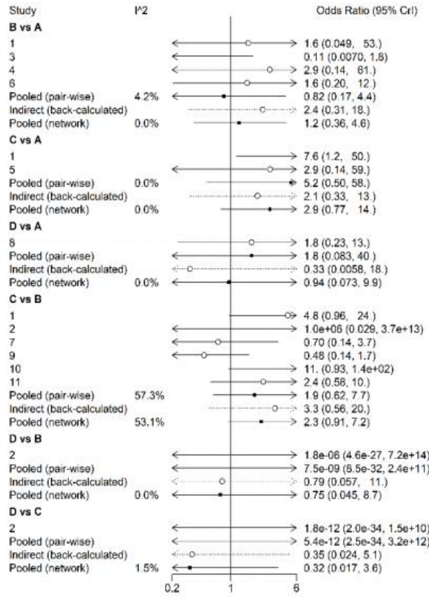

(6) IVH or PVL

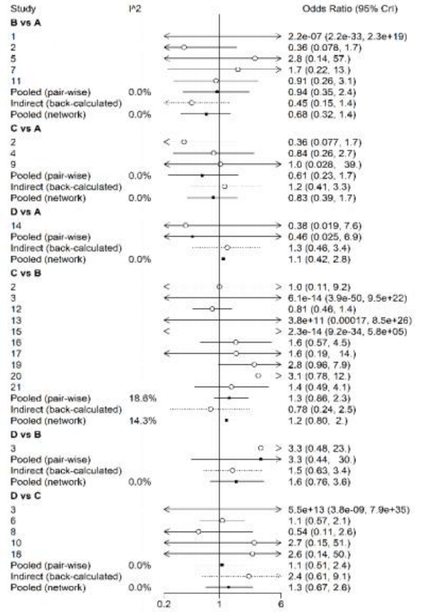

(7) ROP

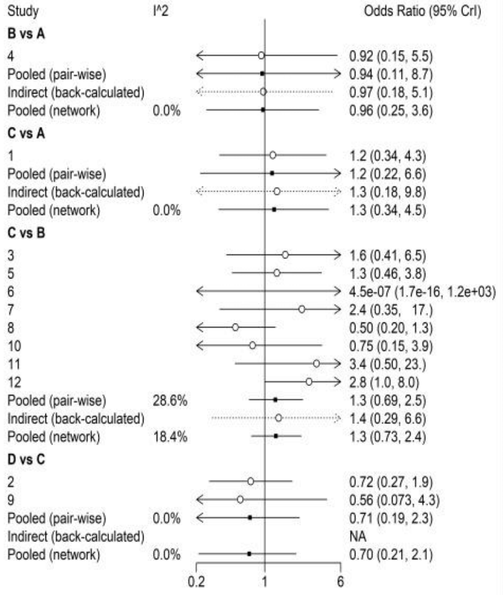

(8) NEC

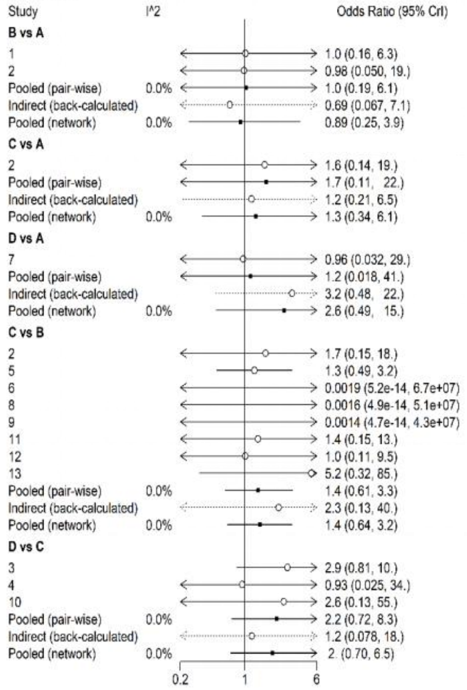

(9) Death

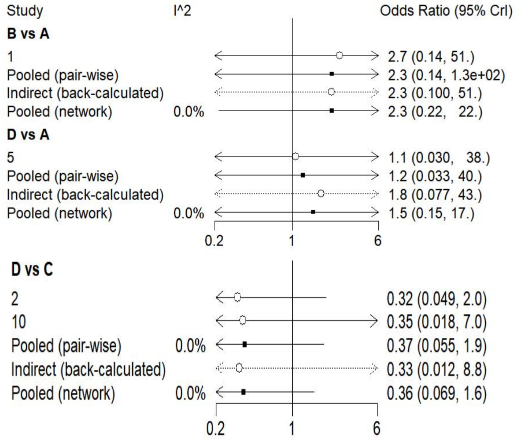

Supplement: Supplementary file 1 [file Datasheet1.pdf]
